# Dermatologic outcomes associated with glucagon-like peptide-1 receptor agonists in patients with type 2 diabetes: a large-scale target trial emulation

**DOI:** 10.3389/fendo.2026.1801203

**Published:** 2026-04-13

**Authors:** Yi-Lun Chiang, Tzu-Hao Li, Yu-Wei Fang, Ya-Fang Liu, Jennifer Wu, Ming-Hsien Tsai

**Affiliations:** 1 Division of Endocrinology, Department of Internal Medicine, Shin-Kong Wu Ho-Su Memorial Hospital, Taipei, Taiwan; 2 Division of Rheumatology, Department of Internal Medicine, Shin-Kong Wu Ho-Su Memorial Hospital, Taipei, Taiwan; 3 Division of Nephrology, Department of Internal Medicine, Shin-Kong Wu Ho-Su Memorial Hospital, Taipei, Taiwan; 4 Department of Medicine, Fu Jen Catholic University, New Taipei City, Taiwan; 5 Department of Digital Medicine, Shin-Kong Wu Ho-Su Memorial Hospital, Taipei, Taiwan; 6 Department of Dermatology, Chang Gung Memorial Hospital, Taoyuan, Taiwan

**Keywords:** autoimmune diseases, dipeptidyl peptidase-4 inhibitors, glucagon-like peptide-1 receptor agonists, incretin-based therapy, psoriasis, real-world evidence

## Abstract

**Background:**

While dipeptidyl peptidase-4 inhibitors (DPP-4is) are linked to autoimmune skin diseases, comparative data on glucagon-like peptide-1 receptor agonists (GLP-1 RAs) remain limited.

**Methods:**

We emulated a randomized active-comparator trial using the TriNetX US Collaborative Network. Adults with type 2 diabetes (T2DM) initiating GLP-1 RAs or DPP-4is between January 1, 2018, and December 31, 2022, were identified, excluding prior users of either class. Balanced cohorts (n=169,630 each) were created using 1:1 propensity score matching. To address protopathic bias, outcomes occurring within 3 months after drug initiation were not considered. Patients were followed for up to 4 years for newly diagnosed autoimmune or inflammatory skin diseases. Cox regression estimated hazard ratios (HRs) and 95% confidence intervals (CIs).

**Results:**

Compared with DPP-4i initiation, GLP-1 RA initiation was associated with a higher risk of incident psoriasis (HR 1.19, 95% CI 1.11–1.28) and lower risks of pemphigus (HR 0.32, 95% CI 0.16–0.63) and bullous pemphigoid (HR 0.61, 95% CI 0.43–0.87). No significant differences were observed for other inflammatory or autoimmune skin outcomes after multiple-testing correction. Findings persisted across subgroup and sensitivity analyses.

**Conclusion:**

In a large, real-world study designed to emulate a clinical trial, GLP-1 RAs were linked to increased psoriasis and decreased autoimmune blistering diseases compared with DPP-4is over up to 4 years’ follow-up. These results provide a dermatologic safety context to guide incretin therapy selection and highlight the importance of ongoing skin monitoring.

## Introduction

Incretin-based therapies have become a cornerstone in the management of type 2 diabetes mellitus (T2DM) (1). Among these agents, glucagon-like peptide-1 receptor agonists (GLP-1 RAs) and dipeptidyl peptidase-4 inhibitors (DPP-4is) improve glycemic control through modulation of the incretin pathway (2), albeit via distinct pharmacological mechanisms. GLP-1 RAs enhance insulin secretion, suppress glucagon release, and promote satiety, resulting in effective glucose lowering and weight reduction (3, 4). In addition to their metabolic effects, GLP-1 RAs have demonstrated substantial cardiovascular and renal benefits in patients with T2DM, leading to their widespread and increasing clinical use (5, 6).

Beyond their glucose-lowering properties, GLP-1 RAs have been shown to exert anti-inflammatory and immunomodulatory effects through actions on immune cells and inflammatory signalling pathways. Experimental and clinical studies suggest that GLP-1 signalling may influence cytokine production, immune cell activation, and tissue inflammation (5, 6). These properties have generated interest in the potential role of GLP-1 RAs in inflammatory and autoimmune diseases (7, 8). Case reports and small clinical studies have described improvements in skin diseases such as psoriasis and hidradenitis suppurativa following GLP-1 RA therapy; however, the overall dermatologic safety profile of GLP-1 RAs remains incompletely characterized (9, 10).

In contrast, DPP-4is—another widely used incretin-based therapy—have been consistently associated with dermatologic adverse effects, particularly autoimmune blistering diseases such as bullous pemphigoid (11, 12). Multiple observational studies and pharmacovigilance reports have demonstrated an increased risk of bullous pemphigoid among patients treated with DPP-4is, establishing these agents as an important reference point when evaluating the dermatologic safety of incretin-based treatments. Given the shared incretin pathway but differing biological effects of GLP-1 RAs and DPP-4is, comparative evaluation of their dermatologic outcomes is clinically relevant.

Although rare cutaneous adverse reactions to GLP-1 RAs—including hypersensitivity reactions, eosinophilic panniculitis, bullous pemphigoid, and morbilliform drug eruptions—have been reported (13), population-level evidence linking GLP-1 RA use to specific autoimmune or inflammatory skin diseases remains limited. Recent studies have also raised concerns regarding possible hair loss associated with GLP-1 RA use (14, 15). A recent meta-analysis reported an increased risk of alopecia among GLP-1 RA users but did not identify significant associations with most other dermatologic outcomes (16). More recently, real-world evidence analyses using the TriNetX database have suggested a possible association between GLP-1 RA use and autoimmune diseases, particularly psoriasis (17). However, the impact of GLP-1 RAs on a broader spectrum of dermatologic conditions has not yet been systematically evaluated.

Unlike prior studies that focused on single dermatologic outcomes or non-comparative designs, the present study emulates a target trial to provide comprehensive, comparative dermatologic safety data for GLP-1 receptor agonists versus DPP-4 inhibitors across a broad spectrum of autoimmune and inflammatory skin diseases. This approach aims to inform real-world treatment selection among incretin-based therapies in patients with type 2 diabetes.

## Methods

### Data sources

TriNetX is a federated research network that provides access to de-identified electronic health record (EHR) data from participating healthcare organizations, primarily within the United States. The platform aggregates real-world clinical data, including demographics, diagnoses, procedures, medications, and laboratory measurements, while maintaining patient privacy through data de-identification and secure query-based access. Investigators can define patient cohorts and perform observational analyses using aggregated statistical outputs without direct access to individual-level data. TriNetX has been widely used for real-world evidence studies across multiple medical disciplines and supports pharmacoepidemiologic analyses using methods such as propensity score matching and time-to-event modelling (18).

### Study design and target trial emulation

We designed this study to emulate a hypothetical target trial (19) evaluating the dermatologic outcomes of initiating GLP-1 RAs compared with initiating DPP-4is among adults with T2DM. The target trial framework guided the specification of eligibility criteria, treatment strategies, assignment procedures, follow-up, and outcome assessment (**Supplementary Table 1**). To approximate this target trial using observational data, we implemented a new-user, intention-to-treat, active-comparator cohort design, which is widely recommended for emulating randomized trials in real-world settings. DPP-4is were selected as the active comparator because they are incretin-based therapies with similar clinical indications and treatment timing, thereby reducing confounding by indication (20). However, because DPP-4is have been associated with autoimmune blistering diseases, this comparison was intended to estimate the relative dermatologic safety of GLP-1 RAs versus an alternative incretin-based therapy, rather than to infer an absolute protective effect of GLP-1 RAs.

### Eligibility criteria and cohort assembly

The study population was identified from individuals with healthcare encounters recorded between January 1, 2018, and December 31, 2022. Eligible patients were required to be aged 18 years or older, have a diagnosis of T2DM, and have at least three recorded healthcare visits prior to cohort entry. To satisfy the new-user requirement of the emulated target trial, patients were required to have no exposure to GLP-1 RAs or DPP-4is during the six months preceding cohort entry.

**Figure 1:**
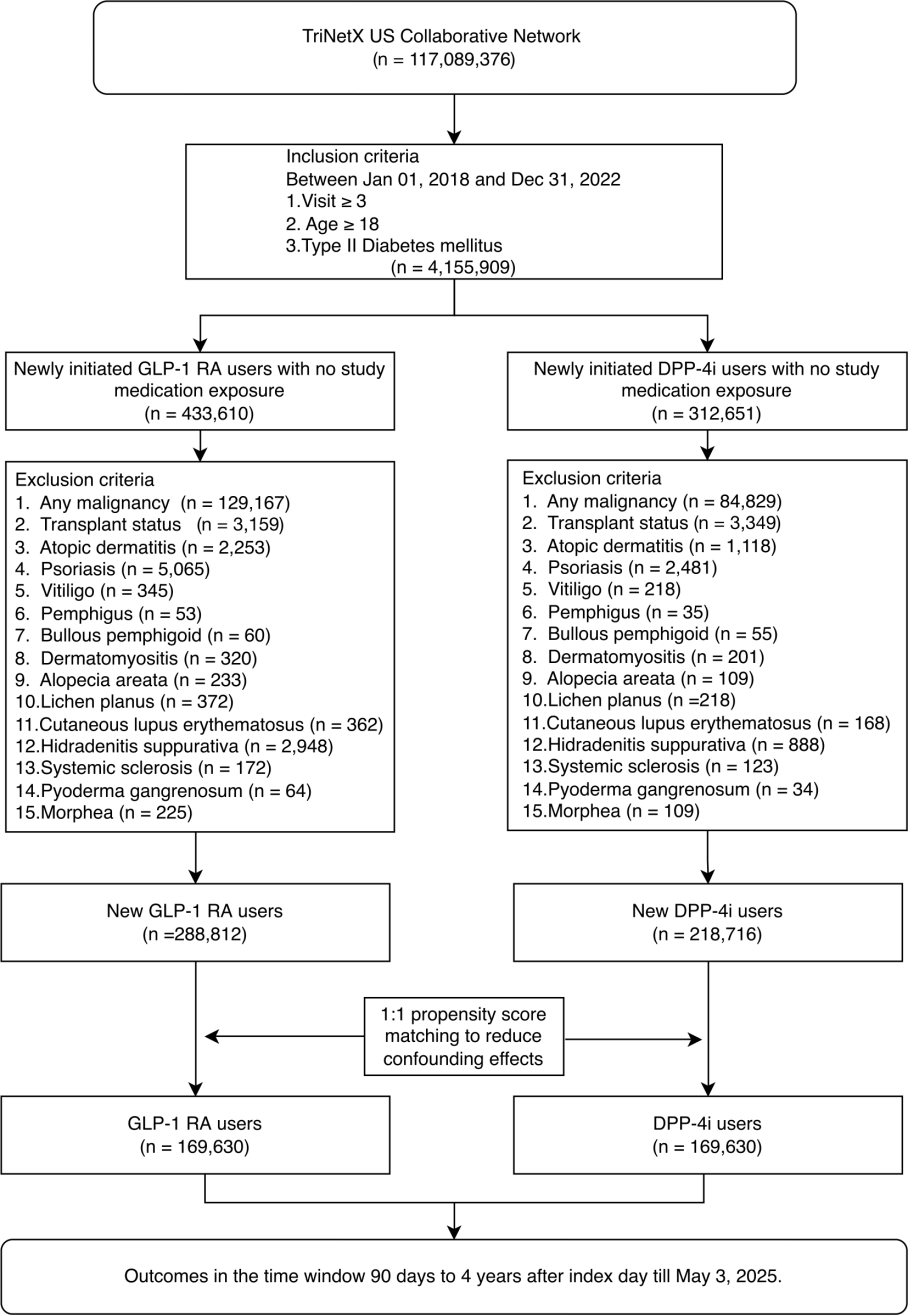
Diagram for creation of study cohorts. GLP1-RA, glucagon-like peptide-1 receptor agonist. DPP-4i, dipeptidyl peptidase 4 inhibitor.

The index date was defined as the date of the first prescription of a GLP-1 RA or DPP-4i during the study period. Patients with a history of malignancy, organ transplantation, or pre-existing dermatologic conditions of interest—including atopic dermatitis, psoriasis, vitiligo, pemphigus, bullous pemphigoid, dermatomyositis, alopecia areata, lichen planus, cutaneous lupus erythematosus, hidradenitis suppurativa, systemic sclerosis, pyoderma gangrenosum, and morphea—were excluded at baseline. After applying these criteria, the final pre-matching cohorts comprised 288,812 new GLP-1 RA users and 218,716 new DPP-4i users. Cohort selection is summarized in **Figure 1**.

In the emulated target trial, treatment strategies were defined as initiation of a GLP-1 RA or initiation of a DPP-4i at baseline. Consistent with an intention-to-treat approach, patients were analyzed according to their initial treatment assignment regardless of subsequent treatment changes.

To mitigate protopathic bias and ensure sufficient biological latency between treatment initiation and the manifestation of dermatologic outcomes, a three-month (90-day) exposure lag period was implemented. We employed a left-truncated follow-up approach (landmark design), where the survival analysis officially commenced at Index Date + 90 days. While all patients were required to meet eligibility criteria at the index date, any dermatologic events occurring during this initial 90-day lag were not counted as outcomes, thereby focusing the analysis on incident cases following established exposure. Patients were followed from this 90-day landmark until the earliest occurrence of a dermatologic outcome, loss to follow-up, death, or the study’s conclusion, with a maximum follow-up duration of four year.

### Propensity score matching

To emulate random treatment assignment in the target trial and control for baseline confounding, propensity score matching (PSM) was performed using the TriNetX analytic environment (21). Propensity scores were estimated using logistic regression models that included baseline demographic characteristics (age, sex, race), comorbidities, medication use, and laboratory measurements (**Table 1**).

**Table 1:** Baseline patient characteristics.

Variables	Before matching			After matching		
	GLP-1 RA	DPP-4i	SMD	GLP-1 RA	DPP-4i	SMD
	(n = 288,812)	(n = 218,716)		(n = 169,630)	(n = 169,630)	
Age at Index (years), Mean ± SD	56.1 ± 12.8	62.3 ± 12.6	0.485	59.8 ± 11.8	59.8 ± 12.4	0.002
BMI(kg/m^2^)	36.5 ± 8.3	32.1 ± 7.5	0.550	34.3 ± 7.9	33.6 ± 7.5	0.011
Systolic BP(mm[Hg])	130.7 ± 17.9	130.1 ± 20.2	0.032	130.8 ± 18.5	130 ± 19.7	0.006
Diastolic BP(mm[Hg])	76.9 ± 11.4	73.7 ± 12.6	0.272	75.9 ± 11.6	74.5 ± 12.5	0.006
Male, n (%)	130,518 (45.2)	110,341 (50.4)	0.105	82,866 (48.9)	82,760 (48.8)	0.001
Race, n (%)						
White people	175,477 (60.8)	123,591 (56.5)	0.086	100,558 (59.3)	100,808 (59.4)	0.003
African American	56,563 (19.6)	42,898 (19.6)	0.001	33,100 (19.5)	33,148 (19.5)	0.001
Asian	9,634 (3.3)	15,067 (6.9)	0.162	7,778 (4.6)	7,494 (4.4)	0.008
Other race	12,411 (4.3)	10,857 (5)	0.032	7,884 (4.6)	7,848 (4.6)	0.001
Lifestyles, n (%)						
Alcohol related disorders	3,717 (1.3)	3,759 (1.7)	0.035	2,553 (1.5)	2,590 (1.5)	0.002
Nicotine dependence	23,776 (8.2)	20,061 (9.2)	0.033	14,543 (8.6)	14,420 (8.5)	0.003
Tobacco use	8,106 (2.8)	6,474 (3)	0.009	4,964 (2.9)	4,986 (2.9)	0.001
Economic circumstances, n (%)						
Persons with potential health hazards related to socioeconomic and psychosocial circumstances	4,677 (1.6)	3,431 (1.6)	0.004	2,683 (1.6)	2,619 (1.5)	0.003
Problems related to employment and unemployment	598 (0.2)	339 (0.2)	0.012	295 (0.2)	289 (0.2)	0.001
Problems related to housing and economic circumstances	1,715 (0.6)	1,465 (0.7)	0.010	1,082 (0.6)	1,052 (0.6)	0.002
Comorbidity, n (%)						
Chronic kidney disease	36,031 (12.5)	41,792 (19.1)	0.183	26,646 (15.7)	26,298 (15.5)	0.006
Atrial fibrillation and flutter	14,260 (4.9)	17,256 (7.9)	0.121	10,738 (6.3)	10,606 (6.3)	0.003
Cardiomyopathy	7,259 (2.5)	7,078 (3.2)	0.043	4,843 (2.9)	4,776 (2.8)	0.002
Cerebrovascular diseases	14,226 (4.9)	17,650 (8.1)	0.128	10,888 (6.4)	10,623 (6.3)	0.006
Dyslipidemia	157,927 (54.7)	118,517 (54.2)	0.010	91,983 (54.2)	91,566 (54)	0.005
Peripheral vascular disease, unspecified	8,636 (3)	8,530 (3.9)	0.050	5,822 (3.4)	5,817 (3.4)	<0.001
Heart failure	20,466 (7.1)	22,115 (10.1)	0.108	14,335 (8.5)	14,259 (8.4)	0.002
Hypertensive diseases	170,841 (59.2)	136,151 (62.3)	0.063	102,701 (60.5)	102,187 (60.2)	0.006
Ischemic heart diseases	40,739 (14.1)	40,869 (18.7)	0.124	28,008 (16.5)	27,749 (16.4)	0.004
Nonrheumatic aortic valve disorders	5,005 (1.7)	5,948 (2.7)	0.067	3,725 (2.2)	3,693 (2.2)	0.001
Other chronic obstructive pulmonary disease	12,717 (4.4)	13,519 (6.2)	0.079	9,129 (5.4)	9,025 (5.3)	0.003
Liver cirrhosis	3,309 (1.1)	2,967 (1.4)	0.019	2,140 (1.3)	2,159 (1.3)	0.001
Disorders of thyroid gland	39,788 (13.8)	27,581 (12.6)	0.034	21,868 (12.9)	21,580 (12.7)	0.005
Gout	7,724 (2.7)	7,187 (3.3)	0.036	5,039 (3)	5,000 (2.9)	0.001
Medication, n (%)						
Insulin	130,350 (45.1)	83,490 (38.2)	0.142	67,644 (39.9)	67,341 (39.7)	0.004
Biguanides	170,314 (59)	137,850 (63)	0.083	106,022 (62.5)	105,251 (62)	0.009
SGLT2i	59,787 (20.7)	30,500 (13.9)	0.179	28,715 (16.9)	27,860 (16.4)	0.014
Sulfonylureas	65,012 (22.5)	67,912 (31.1)	0.194	46,993 (27.7)	46,606 (27.5)	0.005
Thiazolidinediones	14,644 (5.1)	12,191 (5.6)	0.022	9,368 (5.5)	9,310 (5.5)	0.001
Alpha glucosidase inhibitors	743 (0.3)	940 (0.4)	0.029	554 (0.3)	543 (0.3)	0.001
Beta-blocker	90,574 (31.4)	86,347 (39.5)	0.170	60,931 (35.9)	60,495 (35.7)	0.005
Renin-angiotensin system blockade	154,284 (53.4)	126,490 (57.8)	0.089	95,964 (56.6)	95,612 (56.4)	0.004
Calcium channel blockers	66,122 (22.9)	64,347 (29.4)	0.149	44,651 (26.3)	44,535 (26.3)	0.002
Diuretics	101,168 (35)	81,130 (37.1)	0.043	61,893 (36.5)	61,783 (36.4)	0.001
Lipid modifying agents	173,215 (60)	146,238 (66.9)	0.143	109,827 (64.7)	109,072 (64.3)	0.009
Corticosteroids for systemic use	89,956 (31.1)	66,073 (30.2)	0.020	51,649 (30.4)	51,354 (30.3)	0.004
Antihistamine	67,695 (23.4)	52,073 (23.8)	0.009	40,192 (23.7)	39,882 (23.5)	0.004
Antiepileptics	72,303 (25)	52,675 (24.1)	0.022	41,952 (24.7)	41,761 (24.6)	0.003
Antineoplastic and immunomodulating agents	21,920 (7.6)	13,662 (6.2)	0.053	11,227 (6.6)	11,071 (6.5)	0.004
Laboratory, Mean ± SD						
eGFR (mL/min/1.73m^2^)	80.9 ± 29.5	73.5 ± 32.2	0.240	76.5 ± 29	76.9 ± 31.9	0.005
Sodium(mmol/L)	138.1 ± 3	138.1 ± 3.4	0.016	138.2 ± 3.1	138.1 ± 3.3	0.042
Potassium(mmol/L)	4.3 ± 0.5	4.3 ± 0.5	0.004	4.3 ± 0.5	4.2 ± 0.5	0.008
Calcium (mg/dL)	9.4 ± 0.6	9.3 ± 0.7	0.155	9.4 ± 0.6	9.3 ± 0.6	0.008
Phosphate (mg/dL)	3.5 ± 0.9	3.6 ± 1	0.042	3.5 ± 0.9	3.6 ± 1	0.045
UACR (mg/g)	151.9 ± 541.4	165 ± 630.5	0.022	163.6 ± 574.6	150.5 ± 604.7	0.022
HDL-C (mg/dL)	41.4 ± 15.3	40.8 ± 17.1	0.036	41.7 ± 15.6	40.5 ± 16.8	0.005
LDL-C (mg/dL)	92.8 ± 38.9	89.7 ± 39.6	0.078	89.9 ± 38.6	91.3 ± 39.7	0.005
Triglyceride (mg/dL)	197.9 ± 199.4	182.4 ± 176.9	0.082	190.7 ± 187.6	188.3 ± 187	0.005
Hemoglobin (g/dL)	13.5 ± 1.9	12.9 ± 2.2	0.293	13.4 ± 2	13.1 ± 2.1	0.006
Hemoglobin A1c (%)	8.6 ± 2.2	8.3 ± 2.1	0.138	8.5 ± 2.1	8.4 ± 2.1	0.010
Albumin (g/dL)	31.6 ± 37.3	30.2 ± 45.1	0.035	30.4 ± 40.2	30.8 ± 41.6	0.006
AST (U/L)	4.1 ± 0.5	4 ± 0.6	0.221	4.1 ± 0.5	4 ± 0.6	0.007
ALT (U/L)	26.3 ± 41.8	27.2 ± 47.5	0.020	26.1 ± 43.8	26.9 ± 43.7	0.006

GLP-1 RA, glucagon-like peptide-1 receptor agonist; DPP-4i, dipeptidyl peptidase 4 inhibitors; SMD, standardized mean difference; BP, blood pressure; BMI, body mass index; SGLT2i: sodium-glucose transport 2 inhibitors; eGFR, estimated glomerular filtration rate; UACR: microalbumin/creatinine in urine; HDL-C, high density lipoprotein cholesterol; LDL-C, low density lipoprotein cholesterol; AST, aspartate aminotransferase; ALT, alanine aminotransferase.

Patients were matched 1:1 using a greedy nearest-neighbor algorithm with a caliper width of 0.1, based on the pooled standard deviation of the logit of the propensity score. Covariate balance between treatment groups was assessed using standardized mean differences (SMDs), with values <0.1 indicating adequate balance (22). Detailed diagnostic and procedural codes used for covariate definition are provided in **Supplementary Method S1**, and additional details of the matching procedure are presented in **Supplementary Method S2**.

### Study outcomes

The clinical study assessed a variety of dermatological outcomes to compare the effects of GLP-1 RA versus DPP-4i treatments, specifically monitoring for psoriasis (), pemphigus, bullous pemphigoid, and atopic dermatitis. The analysis also included autoimmune and inflammatory conditions such as vitiligo, dermatomyositis, alopecia areata, lichen planus, and cutaneous lupus erythematosus. Furthermore, researchers tracked the incidence of hidradenitis suppurativa, systemic sclerosis, pyoderma gangrenosum, and morphea among the patient groups. Outcomes were identified using International Classification of Diseases (ICD) diagnostic codes, as detailed in **Supplementary Method S1**. Specifically, outcomes were defined by the presence of at least one ICD code recorded in the clinical records during the follow-up period.

### Sensitivity analyses

We conducted prespecified sensitivity analyses to evaluate the robustness of the target trial emulation and to probe key threats to validity, including residual confounding, reverse causation (protopathic bias), and time-related biases. First, we analyzed positive control outcomes—major adverse cardiovascular events (MACE) (23) and major adverse kidney events (MAKE) (24)—given the established cardioprotective and renoprotective effects of GLP-1 RAs in T2DM; observing associations in the expected direction would support adequate confounding control. We also evaluated negative control outcomes (glaucoma and bone fracture), which are not plausibly affected by incretin-based therapies, to detect potential systematic bias such as unmeasured confounding or differential healthcare utilization.

To further reduce protopathic bias, we repeated the primary analysis after extending the exposure lag from 3 to 6 months. We examined sensitivity to confounding-model specification by fitting alternative propensity score models with stepwise covariate inclusion. To assess the influence of follow-up choices and potential informative censoring, we repeated analyses across alternative follow-up windows. Finally, to evaluate robustness to comparator choice and potential channeling, we repeated analyses using additional active comparators (insulin, biguanides, sodium-glucose transport 2 inhibitors [SGLT2i], and thiazolidinediones).

To quantify sensitivity to unmeasured confounding, we additionally calculated E-values for primary associations, reporting values for the point estimate and the 95% confidence limit closest to the null (25).

### Statistical analysis

Baseline characteristics were summarized using counts and percentages for categorical variables and means with standard deviations for continuous variables. Event-free survival was estimated using Kaplan–Meier methods, and between-group differences were assessed using the log-rank test. Associations between treatment strategies and dermatologic outcomes were evaluated using Cox proportional hazards regression models, with hazard ratios (HRs) and 95% confidence intervals (CIs) reported. The proportional hazards assumption was assessed using the generalized Schoenfeld method implemented within the TriNetX platform.

Absolute risk reduction (ARR) was calculated as the difference in Kaplan-Meier survival probabilities between the GLP-1 RA and DPP-4i cohorts at each 2-year time point, providing a clinically interpretable measure of absolute treatment benefit or harm over time. The number needed to treat (NNT) was calculated as the reciprocal of the ARR, representing the number of patients who would need to receive GLP-1 RAs instead of DPP-4is to prevent one outcome event during the study period. When GLP-1 RAs were associated with higher risk, the number needed to harm (NNH) was calculated as the reciprocal of the absolute risk increase, representing the number of patients who would need to be treated for one additional event to occur.

To account for multiple hypothesis testing across dermatologic outcomes, P values were adjusted using the Benjamini–Hochberg false discovery rate procedure (26) with a predefined false discovery rate of 0.05. Subgroup analyses were conducted after repeating PSM within each subgroup, and between-subgroup heterogeneity was assessed using Cochran’s Q test and I² statistic under a random-effects model (27) as a proxy for p-value for interaction, which is unavailable in TriNetX. Missing data were not imputed. All analyses were performed using the TriNetX analytic environment, with supplementary analyses conducted using Python and R (version 3.4.4). A two-sided P value <0.05 was considered statistically significant.

## Results

### Baseline characteristics

Before propensity score matching, substantial differences were observed between patients initiating GLP-1 RAs and those initiating DPP-4is (**Table 1**). Compared with DPP-4i users, GLP-1 RA users were younger (mean age 56.1 vs 62.3 years), had a higher body mass index (36.5 vs 32.1 kg/m²), and were less likely to be male (45.2% vs 50.4%). GLP-1 RA users also had a lower prevalence of chronic kidney disease, atrial fibrillation, and ischemic heart disease, but a higher prevalence of insulin use at baseline.

After 1:1 propensity score matching, 169,630 patients were included in each treatment group. Baseline demographic characteristics, comorbidities, medication use, and laboratory values were well balanced between groups, with SMDs below 0.1 for all covariates, indicating adequate balance consistent with the assumptions of the emulated target trial (**Table 1**).

### Dermatologic outcomes: GLP-1 RA versus DPP-4i use

The mean follow-up duration was 1,080 ± 454 days in the GLP-1 RA group and 1,121 ± 498 days in the DPP-4i group (**Supplementary Figure 1**). Kaplan–Meier curves show 4-year event-free survival for three skin outcomes after matching GLP-1 RA vs DPP-4i initiators (**Supplementary Figure 2**). Psoriasis incidence is higher with GLP-1 RAs (**Supplementary Figure 2A**; log-rank p<0.001). Pemphigus and bullous pemphigoid are lower with GLP-1 RAs (**Supplementary Figures 2B, C**; p=0.001 and 0.005). Shaded areas are CIs; curves separate early and remain apart despite low absolute event rates.

In **Table 2**, after PSM, initiation of GLP-1 RA therapy was associated with a modestly higher incidence of psoriasis compared with initiation of DPP-4i therapy (hazard ratio [HR] 1.19, 95% confidence interval [CI] 1.11–1.28) (**Table 2**). In contrast, GLP-1 RA initiation was associated with lower incidences of pemphigus (HR 0.32, 95% CI 0.16–0.63) and bullous pemphigoid (HR 0.61, 95% CI 0.43–0.87). No statistically significant differences were observed between GLP-1 RA and DPP-4i users for atopic dermatitis, vitiligo, dermatomyositis, alopecia areata, lichen planus, cutaneous lupus erythematosus, hidradenitis suppurativa, systemic sclerosis, pyoderma gangrenosum, or morphea.

**Table 2:** Major clinical outcomes after matching.

Clinical outcomes	GLP-1 RA users (n = 169,630)		DPP-4i users (n = 169,630)		GLP-1 RA vs. DPP-4i users		
	Events (n)	Risk (%)	Events (n)	Risk (%)	HR (95% CI)	P value	FDR-adjusted p value
Psoriasis	1,597	0.941	1,390	0.819	1.19 (1.11–1.28)	<0.001	0.005
Pemphigus	11	0.006	36	0.021	0.32 (0.16–0.63)	0.001	0.006
Bullous pemphigoid	48	0.028	82	0.048	0.61 (0.43–0.87)	0.005	0.016
Atopic dermatitis	948	0.559	897	0.529	1.11 (1.01–1.21)	0.031	0.062
Vitiligo	131	0.077	128	0.075	1.06 (0.83–1.35)	0.651	0.744
Dermatomyositis	79	0.047	76	0.045	1.07 (0.78–1.46)	0.692	0.744
Alopecia areata	91	0.054	85	0.05	1.13 (0.84–1.52)	0.415	0.544
Lichen planus	177	0.104	160	0.094	1.15 (0.93–1.43)	0.193	0.302
Cutaneous lupus erythematosus	109	0.064	107	0.063	1.03 (0.79–1.35)	0.825	0.858
Hidradenitis suppurativa	377	0.222	360	0.212	1.10 (0.95–1.27)	0.192	0.302
Systemic sclerosis	86	0.051	91	0.054	0.97 (0.72–1.30)	0.829	0.858
Pyoderma gangrenosum	21	0.012	24	0.014	0.94 (0.52–1.69)	0.833	0.858
Morphea	82	0.048	66	0.039	1.31 (0.95–1.81)	0.102	0.192

GP-1 RA, glucagon-like peptide-1 receptor agonist; DPP-4i, Dipeptidyl peptidase 4 inhibitors; HR, hazard ratio; 95% CI, 95% confidence interval, FDR, Benjamini-Hochberg false discovery rate.

After adjustment for multiple comparisons using the Benjamini–Hochberg procedure, only the associations with psoriasis, pemphigus, and bullous pemphigoid remained statistically significant.

### Clinical implication

Absolute risk analysis showed that initiating GLP-1 RAs rather than DPP-4is was associated with a small increase in psoriasis risk and a reduced risk of autoimmune blistering diseases (**Table 3**). At 2 years, GLP-1 RA use was associated with an absolute risk increase for psoriasis of 0.05%, rising to 0.08% at 4 years. This corresponded to a NNH of 1,198, indicating that approximately one additional case of psoriasis would occur for every 1,200 patients treated with GLP-1 RAs instead of DPP-4is.

**Table 3:** Clinical outcomes in patients with type 2 diabetes and psoriasis.

Clinical outcomes	After propensity score matching^$^ within 4-year follow-up					
	GLP-1 RA user (n = 3,552)		DPP-4i user (n = 3,552)		GLP-1 RA and DPP-4i	
	Events (n)	Risk (%)	Events (n)	Risk (%)	HR (95%CI)	P value
^#^MACE	738	20.777	860	24.212	0.91 (0.83–1.01)	0.069
^%^MAKE	580	16.329	711	20.017	0.88 (0.79–0.98)	0.021^†^
Mortality	140	3.941	214	6.025	0.74 (0.6–0.92)	0.006
Hospitalization	1,031	29.026	1,193	33.587	0.91 (0.84–0.99)	0.024
AMI	158	4.448	196	5.518	0.9 (0.73–1.11)	0.337^†^
Heart failure	531	14.949	595	16.751	0.95 (0.84–1.06)	0.360
Stroke	148	4.167	164	4.617	1 (0.8–1.25)	1.000
Sepsis	268	7.545	341	9.600	0.86 (0.73–1.01)	0.061^†^
ESKD requiring dialysis	73	2.055	112	3.153	0.71 (0.53–0.95)	0.022^†^
ED visiting	1,207	33.981	1,230	34.628	1.05 (0.97–1.14)	0.228

^$^Propensity score matching was performed using the parameters in Method S1.

^#^MACE included myocardial infarction, stroke, and death.

^%^MAKE included acute kidney injury, end stage of kidney disease, dialysis, and death.

^†^This indicate the proportional hazard assumption is violated.

GLP-1 RA, glucagon-like peptide-1 receptor agonists; DPP-4i, dipeptidyl peptidase-4 inhibitor; HR, hazard ratio; CI, confidence interval; MACE, major adverse cardiovascular events; MAKE, major adverse kidney events; AMI, acute myocardial infarction; ESKD, end-stage kidney disease; ED, emergency department.

In contrast, GLP-1 RAs showed a protective effect against rare bullous diseases. At 4 years, ARR was 0.03% for bullous pemphigoid, corresponding to a NNT of 3,796, and 0.02% for pemphigus, corresponding to an NNT of 5,257. Although these absolute differences were small, they provide important clinical context by showing that the relative safety signals observed in the study corresponded to low absolute event rates in routine practice.

### Subgroup analyses

**Figure 2:**
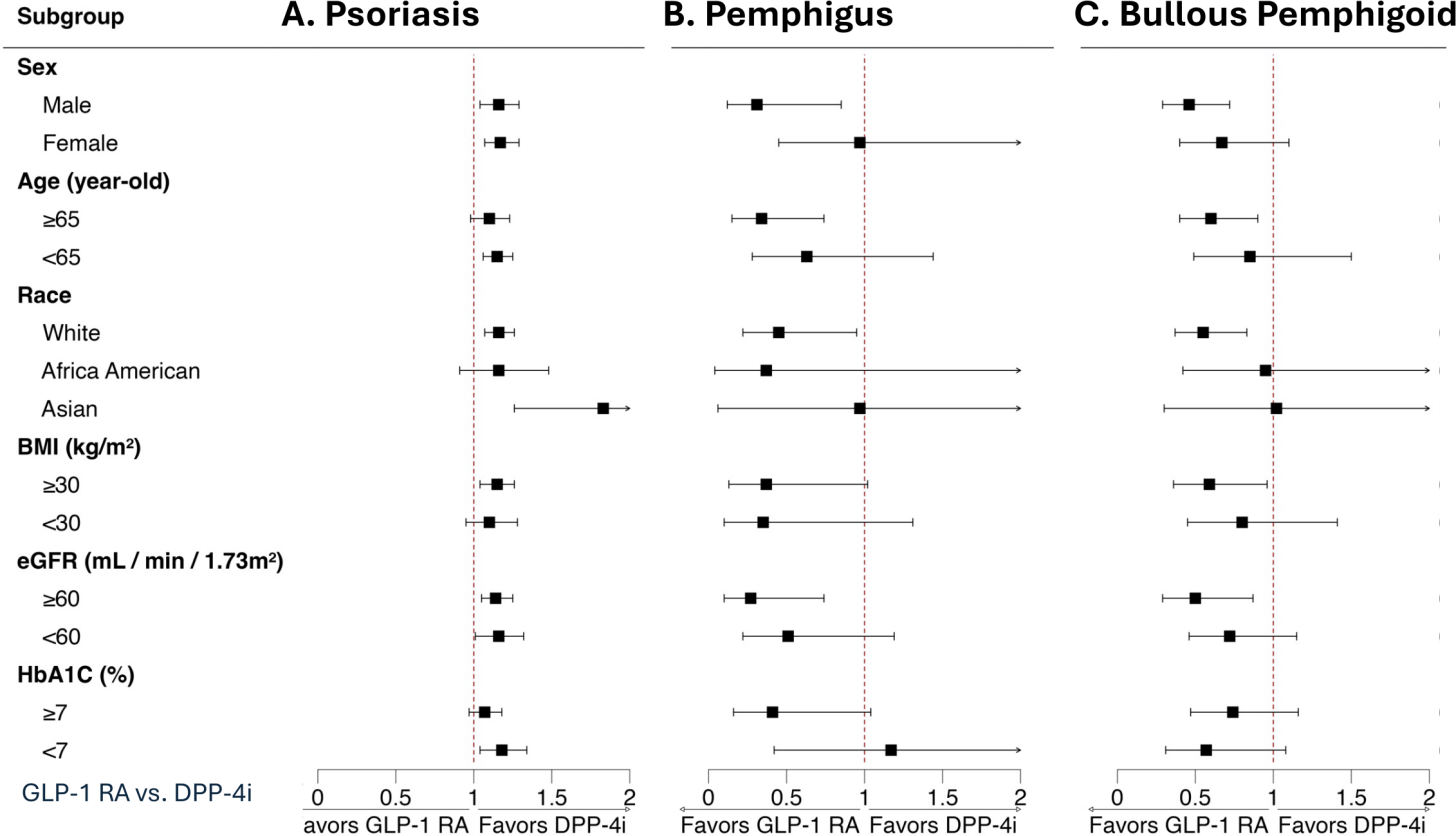
Subgroup analysis. Outcomes of **(A)** psoriasis, **(B)** pemphigus, and **(C)** bullous pemphigoid between patients receiving GLP-1 RAs versus DPP-4is were evaluated. Subgroup analyses were conducted after repeating 1:1 propensity score matching within each specific subgroup to ensure baseline covariate balance. Heterogeneity between subgroups was assessed using Cochran's Q test (details are presented in **Supplementary Figures S3**-**S5**). GLP1-RA, glucagon-like peptide-1 receptor agonist. DPP-4i, dipeptidyl peptidase 4 inhibitor.

**Figure 2** presents subgroup analyses of matched GLP-1 RA versus DPP-4i initiators for psoriasis, pemphigus, and bullous pemphigoid. For psoriasis (**Figure 2A**), HRs generally favored DPP-4i, indicating a modestly higher psoriasis incidence with GLP-1 RAs across sex, age (<65 and ≥65 years), race, BMI strata, kidney function (eGFR <60 and ≥60 ml/mini/1.73m2), and glycemic control (HbA1c <7 and ≥7%), with no evidence of meaningful heterogeneity. For pemphigus and bullous pemphigoid (**Figures 2B, C**), estimates favored GLP-1 RAs across subgroups, consistent with lower risks. Confidence intervals overlapped in some strata, but directionality was stable, supporting robustness of the primary findings. (The details of subgroup analysis are shown in **Supplementary Figures S3**-**S5**).

### Sensitivity analyses

E-values (**Table 2**) supported moderate-to-strong robustness of the observed associations to potential unmeasured confounding. For psoriasis (HR 1.19), an unmeasured confounder would require risk-ratio associations of at least 1.67 with both GLP-1 RA initiation (vs DPP-4i) and incident psoriasis to fully explain the observed effect. Robustness was greater for inverse associations, with E-values of 5.70 for pemphigus (HR 0.32) and 2.66 for bullous pemphigoid (HR 0.61), indicating that comparatively strong residual confounding would be necessary to nullify these findings.

Sensitivity analyses were concordant with the primary results. As expected, positive control analyses showed lower risks of major adverse cardiovascular events and major adverse kidney events among GLP-1 RA initiators relative to DPP-4i initiators, whereas negative control outcomes (glaucoma and bone fracture) did not differ between groups (**Supplementary Table 2**). Extending the exposure lag from 3 to 6 months yielded materially similar estimates (**Supplementary Table 3**). Results were also stable across alternative propensity score specifications with stepwise covariate adjustment (**Supplementary Table 4**) and across prespecified follow-up windows (**Supplementary Table 5**).

### Comparison with other glucose-lowering medications

**Figure 3:**
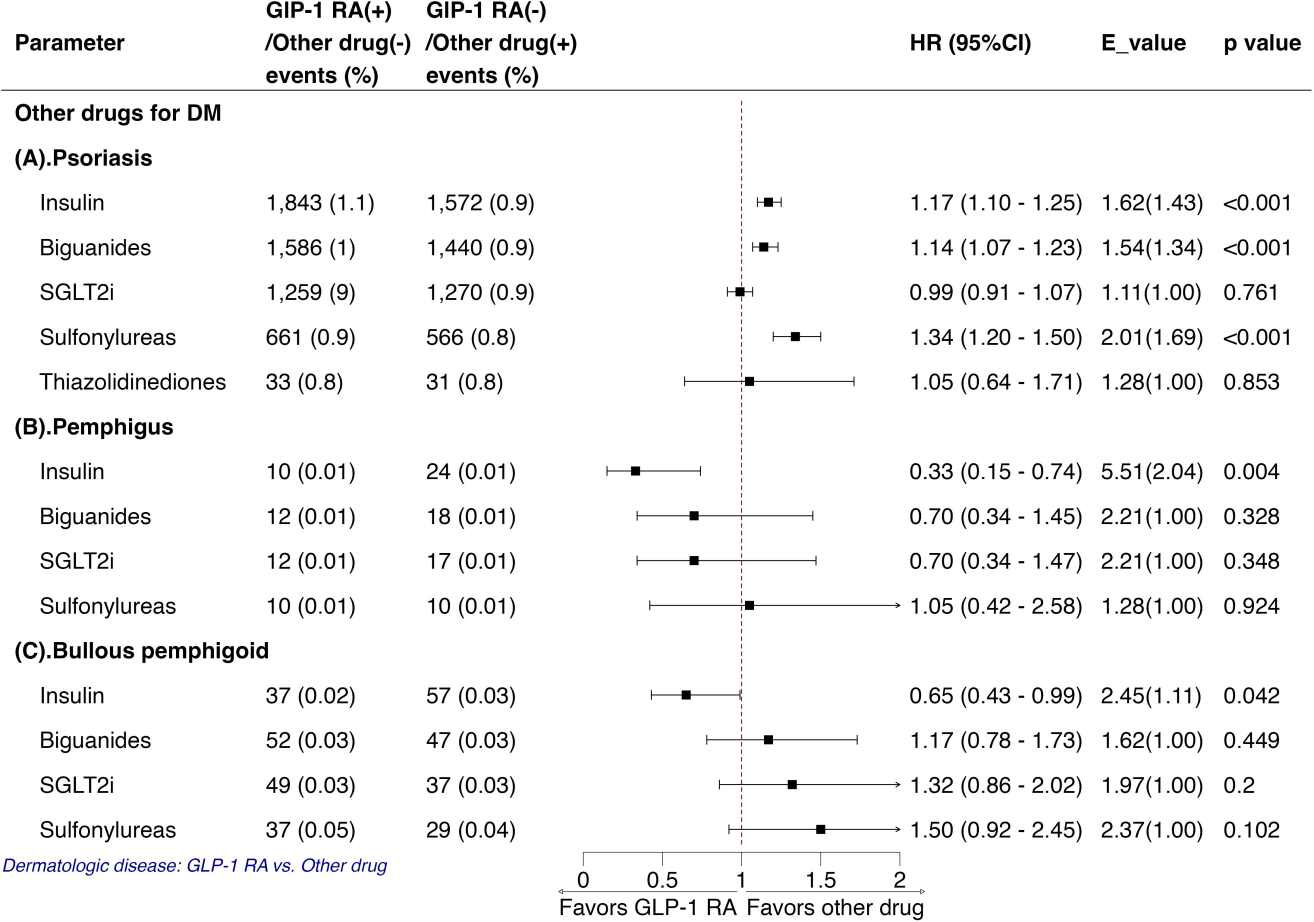
Analysis of the effects of GLP-1 RAs versus other antidiabetic medications on the outcome of **(A)** psoriasis, **(B)** pemphigus, and **(C)** bullous pemphigoid Each comparison represents an independent target trial emulation using a new-user design with 1:1 propensity score matching between GLP-1 RA users and the specific comparator cohort. DM, diabetes mellitus; GLP1-RA, glucagon-like peptide-1 receptor agonist. DPP-4i, dipeptidyl peptidase 4 inhibitors; SGLT2i, sodium-glucose cotransporter-2 inhibitors.

**Figure 3** compares the risk of dermatologic diseases in patients with diabetes treated with GLP-1 RA versus other antidiabetic drugs. GLP-1 RA use was associated with a higher risk of psoriasis compared with insulin (HR 1.17, 95% CI 1.10–1.25), biguanides (HR 1.14, 95% CI 1.07–1.23), and sulfonylureas (HR 1.34, 95% CI 1.20–1.50), while no significant differences were observed versus SGLT2i (HR 0.99, 95% CI 0.91–1.07) or thiazolidinediones (HR 1.05, 95% CI 0.64–1.71) (**Figure 3A**). In contrast, GLP-1 RA use was associated with a significantly lower risk of pemphigus compared with insulin (HR 0.33, 95% CI 0.15–0.74), with no significant differences versus other oral agents (**Figure 3B**). Similarly, GLP-1 RA use was linked to a reduced risk of bullous pemphigoid compared with insulin (HR 0.65, 95% CI 0.43–0.99), while comparisons with other antidiabetic drugs were not statistically significant (**Figure 3C**).

## Discussion

In this large-scale real-world cohort study emulating a target trial, we evaluated the comparative dermatologic outcomes associated with initiation of GLP-1 receptor agonists versus DPP-4 inhibitors among patients with T2DM. We found that GLP-1 RA use was associated with a modestly higher incidence of psoriasis and lower incidences of pemphigus and bullous pemphigoid compared with DPP-4i use, while no increased risk was observed for most other autoimmune or inflammatory skin diseases. By using an active-comparator incretin-based design and evaluating a broad range of dermatologic outcomes, this study extends prior real-world evidence and provides clinically relevant comparative safety data.

The observed association between GLP-1 RA initiation and a modestly higher incidence of psoriasis warrants careful interpretation. Psoriasis is a chronic immune-mediated inflammatory skin disease driven by dysregulated interactions between keratinocytes and immune cells, particularly involving the interleukin (IL)-23/IL-17 axis and tumor necrosis factor (TNF)-α signaling (28, 29). Although GLP-1 RAs have demonstrated anti-inflammatory effects in experimental and clinical studies, including modulation of cytokine production and immune cell activation (7, 8), their immunologic effects are complex and may vary depending on host susceptibility. Recent real-world evidence analyses have similarly reported an increased incidence of psoriasis among GLP-1 RA users, lending support to our findings (17). Importantly, the absolute incidence of psoriasis in both treatment groups was low, and the observed association should be interpreted as a relative difference rather than a contraindication to GLP-1 RA therapy.

It is important to note that out of the thirteen dermatologic outcomes systematically evaluated in this target trial emulation, only three—psoriasis, pemphigus, and bullous pemphigoid—maintained statistical significance after rigorous adjustment for multiple testing using the Benjamini–Hochberg procedure. While nominal associations were observed for other conditions, such as atopic dermatitis (p = 0.031), these did not meet the predefined false discovery rate threshold (FDR-adjusted p = 0.062) and are therefore considered statistically non-significant. Consequently, our findings do not support an increased risk for the broader spectrum of autoimmune or inflammatory skin diseases beyond the specific associations identified for psoriasis and blistering diseases.

In a nationwide cohort study of patients with psoriasis, GLP-1 RA use was associated with reduced risks of all-cause mortality, cardiovascular events, and psychiatric disorders compared with other glucose-lowering therapies (30). These findings suggest that, despite the modest increase in psoriasis incidence reported in some real-world studies, GLP-1 RAs may offer important systemic benefits in this population. Accordingly, dermatologic safety signals should be interpreted in the context of broader clinical benefit, and an increased psoriasis incidence should not be considered a contraindication to GLP-1 RA therapy. Although GLP-1 RAs were associated with a modestly increased relative risk of psoriasis, the absolute excess risk was small (4-year ARI, 0.08%; NNH, 1,198). This finding should therefore not preclude treatment but may support counseling patients about possible new-onset psoriatic symptoms and routine skin monitoring after initiation.

In contrast, initiation of GLP-1 RAs was associated with substantially lower incidences of pemphigus and bullous pemphigoid compared with DPP-4i initiation. This finding is clinically relevant given the well-established association between DPP-4i use and autoimmune blistering diseases, particularly bullous pemphigoid (11, 12). DPP-4 is involved in immune regulation, antigen presentation, and T-cell activation, and pharmacologic inhibition of DPP-4 has been hypothesized to promote autoantibody formation against components of the dermal–epidermal junction (11, 31). The lower incidence of blistering diseases observed among GLP-1 RA users likely reflects, at least in part, the absence of DPP-4i exposure rather than a direct protective effect of GLP-1 RAs. Nevertheless, these findings provide clinically meaningful comparative safety information when selecting incretin-based therapies for patients at increased risk of autoimmune skin disorders, although the absolute risk reductions were small at 4 years (0.03% for bullous pemphigoid and 0.02% for pemphigus).

the lower relative risks of bullous pemphigoid and pemphigus compared with DPP-4is provide useful comparative safety information when selecting incretin-based therapy, particularly in patients with a history of autoimmune blistering disease or those considered at higher dermatologic risk. Overall, these results support individualized treatment decisions that balance the well-established cardiometabolic benefits of GLP-1 RAs against a small absolute increase in psoriasis risk.

From a clinical perspective, these findings support the continued use of GLP-1 RAs in patients with T2DM, including those who may be at increased risk for autoimmune blistering diseases. Although a modest increase in psoriasis incidence was observed, the absolute risk remained low and should be interpreted in the context of the well-established cardiometabolic benefits of GLP-1 RAs. Rather than avoiding GLP-1 RA therapy, clinicians should maintain dermatologic awareness and monitor for new-onset skin disease, particularly during early treatment.

Several methodological strengths support the validity of this study. We employed a new-user, active-comparator design guided by target trial emulation principles, which is recommended to minimize immortal time bias and confounding by indication in observational drug safety research. PSM achieved a good balance across measured covariates, and the inclusion of positive and negative control outcomes further supports internal validity. By leveraging a large, heterogeneous electronic health record database, this study complements randomized clinical trial data, which are often underpowered to evaluate rare dermatologic outcomes.

Several limitations of this study should be acknowledged. First, as an observational study based on EHR data, residual confounding due to unmeasured or incompletely measured variables cannot be fully excluded, despite the use of a new-user, active-comparator design and rigorous PSM. Second, the dermatologic outcomes were determined exclusively using ICD diagnostic codes rather than specialist-adjudicated diagnoses or histopathologic confirmation. Although this approach is commonly used in large-scale real-world studies, misclassification of outcomes is possible, particularly for conditions with heterogeneous clinical presentations such as psoriasis or atopic dermatitis. In addition, information on disease severity, extent of skin involvement, and treatment response was unavailable, precluding analyses of dose–response relationships or severity-specific outcomes. However, consistent sensitivity analyses in this study help mitigate such bias. Third, differential healthcare utilization may have contributed to detection bias. Patients initiating GLP-1 RAs may have more frequent clinical encounters due to closer metabolic monitoring, weight management visits, or follow-up related to gastrointestinal adverse effects. Increased healthcare contact could lead to higher diagnostic ascertainment of dermatologic conditions, including psoriasis, compared with patients treated with DPP-4i. Fourth, medication exposure was defined based on prescription records, and information on actual drug adherence, persistence, dose escalation, or treatment discontinuation was not available. Although an intention-to-treat framework was used to emulate the target trial, treatment switching or discontinuation during follow-up could have attenuated or biased observed associations. Fifth, although the TriNetX US Collaborative Network includes data from multiple healthcare systems and a large, diverse patient population, the findings may not be fully generalizable to populations outside the United States or to healthcare settings with different prescribing practices, genetic backgrounds, or access to dermatologic care. Sixth, some outcomes—particularly pemphigus—had very small numbers of events after matching. Although point estimates suggested lower risks among GLP-1 RA users, these results may be unstable because sparse-event analyses can produce imprecise effect estimates. Therefore, these findings should be interpreted cautiously and considered hypothesis-generating until confirmed in studies with larger event numbers. Finally, while the study was designed to emulate a target trial, causal inference remains limited by the observational nature of the data. The results should therefore be interpreted as associations rather than evidence of causality. Future prospective studies and mechanistic investigations are needed to further clarify the biological pathways linking incretin-based therapies and dermatologic outcomes.

## Conclusion

In this large real-world study emulating a target trial, initiation of GLP-1 RA therapy among patients with T2DM was associated with a modestly higher incidence of psoriasis and lower incidences of pemphigus and bullous pemphigoid compared with DPP-4i therapy, while no increased risk was observed for most other autoimmune or inflammatory skin diseases. These findings provide comparative dermatologic safety evidence to inform incretin-based therapy selection in routine clinical practice.

## Data Availability

The data analyzed in this study were obtained from the TriNetX research network and are not publicly available due to data use restrictions. The datasets are not included in the article or **Supplementary Material**. Access to TriNetX data is available to qualified researchers through TriNetX (TriNetX, LLC) subject to a data use agreement and applicable institutional requirements. Requests to access these datasets should be directed to Ming-Hsien Tsai, chaosmyth.tw@gmail.com.
