# Supplementary material for: Dermatologic outcomes associated with glucagon-like peptide-1 receptor agonists in patients with type 2 diabetes: a large-scale target trial emulation
Source: Front Endocrinol (Lausanne). 2026 Apr 13;17:1801203. doi: 10.3389/fendo.2026.1801203 (PMC13111083; doi:10.3389/fendo.2026.1801203)
Supplement: Supplementary file 1 [file DataSheet1.docx]

Supplementary Material

[**Method S1.** Code for cohorts and outcomes, and baseline data 2](#_Toc220365563)

[**Method S2.** The details of propensity score matching in the TriNetX platform 15](#_Toc220365564)

[**Figure S1.** The distribution of follow-up time 20](#_Toc220365565)

[**Figure S2.** Kaplan-Meier cumulative event-free plots 21](#_Toc220365566)

[**Figure S3.** Subgroup on the outcome of Psoriasis 22](#_Toc220365567)

[**Figure S4.** Subgroup on the outcome of pemphigus 23](#_Toc220365568)

[**Figure S5.** Subgroup on the outcome of bullous pemphigoid 24](#_Toc220365569)

[**Table S1.** Target trail emulation 25](#_Toc220365570)

[**Table S2.** The positive and negative outcome control 27](#_Toc220365571)

[**Table S3.** Sensitivity test by extending the index date by 6 months 28](#_Toc220365572)

[**Table S4.** Models for confounding adjustment 29](#_Toc220365573)

[**Table S5.** Segmenting the follow-up into three periods 30](#_Toc220365574)

**Method S1.** Code for cohorts and outcomes, and baseline data

**Cohort definition**

**Query Criteria for Cohort 1 (query name: GLP-1 RA)**

|  | | | | | |
| --- | --- | --- | --- | --- | --- |
| Group 1 | | | | | |
|  | **Visit >=3** | | | | |
|  | must have |  | visit | TNX:Visit | Visit |
|  | number of instances | | Greater than or equal to 3 instances | | |
|  | date constraint | | The terms in this group occurred at any time | | |
| Group 2 | | | | | |
|  | **Type 2 DM** | | | | |
|  | must have |  | diagnosis | UMLS:ICD10CM:E11 | Type 2 diabetes mellitus (at least 18 years old at event) |
|  | date constraint | | The terms in this group occurred between Jan 1, 2018 and Dec 31, 2022 | | |
| Group 3 | | | | | |
|  | **GLP-1 RA use** | | | | |
|  | must have |  | medication | NLM:ATC:A10BJ | Glucagon-like peptide-1 (GLP-1) analogues |
|  | date constraint | | The terms in this group occurred between Jan 1, 2018 and Dec 31, 2022 | | |
| Group 4 | | | | | |
|  | **Group 4A : GLP-1 RA use** | | | | |
|  | must have |  | medication | NLM:ATC:A10BJ | Glucagon-like peptide-1 (GLP-1) analogues |
|  | date constraint | | The terms in this group occurred between Jan 1, 2018 and Dec 31, 2022 | | |
|  | event relationship | | Any instance of Group 4B occurred within 3 months on or after the first instance of Group 4A | | |
|  | **Group 4B No DPP-4i** | | | | |
|  | cannot have |  | medication | NLM:ATC:A10BH | Dipeptidyl peptidase 4 (DPP-4) inhibitors |
| Group 5 | | | | | |
|  | **Group 5A: GLP-1 RA use** | | | | |
|  | must have |  | medication | NLM:ATC:A10BJ | Glucagon-like peptide-1 (GLP-1) analogues |
|  | date constraint | | The terms in this group occurred between Jan 1, 2018 and Dec 31, 2022 | | |
|  | event relationship | | Any instance of Group 5B occurred within 6 months and 1 day before the first instance of Group 5A | | |
|  | **Group 5B no GLP-1 RA and DPP-4i** | | | | |
|  | cannot have |  | medication | NLM:ATC:A10BJ | Glucagon-like peptide-1 (GLP-1) analogues |
|  |  | or | medication | NLM:ATC:A10BH | Dipeptidyl peptidase 4 (DPP-4) inhibitors |
| Group 6 | | | | | |
|  | **Group 6A : GLP-1 RA use** | | | | |
|  | must have |  | medication | NLM:ATC:A10BJ | Glucagon-like peptide-1 (GLP-1) analogues |
|  | date constraint | | The terms in this group occurred between Jan 1, 2018 and Dec 31, 2022 | | |
|  | event relationship | | Any instance of Group 6B occurred at least 1 day before the first instance of Group 6A | | |
|  | **Group 6B No Neoplasms** | | | | |
|  | cannot have |  | diagnosis | UMLS:ICD10CM:C00-D49 | Neoplasms |
| Group 7 | | | | | |
|  | **Group 7A : GLP-1 RA use** | | | | |
|  | must have |  | medication | NLM:ATC:A10BJ | Glucagon-like peptide-1 (GLP-1) analogues |
|  | date constraint | | The terms in this group occurred between Jan 1, 2018 and Dec 31, 2022 | | |
|  | event relationship | | Any instance of Group 7B occurred at least 1 day before the first instance of Group 7A | | |
|  | **Group 7B No Transplant** | | | | |
|  | cannot have |  | diagnosis | UMLS:ICD10CM:Z94 | Transplanted organ and tissue status |
| Group 8 | | | | | |
|  | **Group 8A : GLP-1 RA use** | | | | |
|  | must have |  | medication | NLM:ATC:A10BJ | Glucagon-like peptide-1 (GLP-1) analogues |
|  | date constraint | | The terms in this group occurred between Jan 1, 2018 and Dec 31, 2022 | | |
|  | event relationship | | Any instance of Group 8B occurred at least 1 day before the first instance of Group 8A | | |
|  | **Group 8B No Psoriasis** | | | | |
|  | cannot have |  | diagnosis | UMLS:ICD10CM:L40 | Psoriasis |
| Group 9 | | | | | |
|  | **Group 9A : GLP-1 RA use** | | | | |
|  | must have |  | medication | NLM:ATC:A10BJ | Glucagon-like peptide-1 (GLP-1) analogues |
|  | date constraint | | The terms in this group occurred between Jan 1, 2018 and Dec 31, 2022 | | |
|  | event relationship | | Any instance of Group 9B occurred at least 1 day before the first instance of Group 9A | | |
|  | **Group 9B No Alopecia areata** | | | | |
|  | cannot have |  | diagnosis | UMLS:ICD10CM:L63 | Alopecia areata |
| Group 10 | | | | | |
|  | **Group 10A : GLP-1 RA use** | | | | |
|  | must have |  | medication | NLM:ATC:A10BJ | Glucagon-like peptide-1 (GLP-1) analogues |
|  | date constraint | | The terms in this group occurred between Jan 1, 2018 and Dec 31, 2022 | | |
|  | event relationship | | Any instance of Group 10B occurred at least 1 day before the first instance of Group 10A | | |
|  | **Group 10B No Vitilito** | | | | |
|  | cannot have |  | diagnosis | UMLS:ICD10CM:L80 | Vitiligo |
| Group 11 | | | | | |
|  | **Group 11A : GLP-1 RA use** | | | | |
|  | must have |  | medication | NLM:ATC:A10BJ | Glucagon-like peptide-1 (GLP-1) analogues |
|  | date constraint | | The terms in this group occurred between Jan 1, 2018 and Dec 31, 2022 | | |
|  | event relationship | | Any instance of Group 11B occurred at least 1 day before the first instance of Group 11A | | |
|  | **Group 11B No Lichen planus** | | | | |
|  | cannot have |  | diagnosis | UMLS:ICD10CM:L43 | Lichen planus |
| Group 12 | | | | | |
|  | **Group 12A : GLP-1 RA use** | | | | |
|  | must have |  | medication | NLM:ATC:A10BJ | Glucagon-like peptide-1 (GLP-1) analogues |
|  | date constraint | | The terms in this group occurred between Jan 1, 2018 and Dec 31, 2022 | | |
|  | event relationship | | Any instance of Group 12B occurred at least 1 day before the first instance of Group 12A | | |
|  | **Group 12B No Pemphigus** | | | | |
|  | cannot have |  | diagnosis | UMLS:ICD10CM:L10 | Pemphigus |
| Group 13 | | | | | |
|  | **Group 13A : GLP-1 RA use** | | | | |
|  | must have |  | medication | NLM:ATC:A10BJ | Glucagon-like peptide-1 (GLP-1) analogues |
|  | date constraint | | The terms in this group occurred between Jan 1, 2018 and Dec 31, 2022 | | |
|  | event relationship | | Any instance of Group 13B occurred at least 1 day before the first instance of Group 13A | | |
|  | **Group 13B No Bullous pemphigoid** | | | | |
|  | cannot have |  | diagnosis | UMLS:ICD10CM:L12.0 | Bullous pemphigoid |
| Group 14 | | | | | |
|  | **Group 14A : GLP-1 RA use** | | | | |
|  | must have |  | medication | NLM:ATC:A10BJ | Glucagon-like peptide-1 (GLP-1) analogues |
|  | date constraint | | The terms in this group occurred between Jan 1, 2018 and Dec 31, 2022 | | |
|  | event relationship | | Any instance of Group 14B occurred at least 1 day before the first instance of Group 14A | | |
|  | **Group 14B No Atopic dermatitis** | | | | |
|  | cannot have |  | diagnosis | UMLS:ICD10CM:L20 | Atopic dermatitis |
| Group 15 | | | | | |
|  | **Group 15A : GLP-1 RA use** | | | | |
|  | must have |  | medication | NLM:ATC:A10BJ | Glucagon-like peptide-1 (GLP-1) analogues |
|  | date constraint | | The terms in this group occurred between Jan 1, 2018 and Dec 31, 2022 | | |
|  | event relationship | | Any instance of Group 15B occurred at least 1 day before the first instance of Group 15A | | |
|  | **Group 15B No Dermatopolymyositis** | | | | |
|  | cannot have |  | diagnosis | UMLS:ICD10CM:M33 | Dermatopolymyositis |
| Group 16 | | | | | |
|  | **Group 16A: GLP-1 RA use** | | | | |
|  | must have |  | medication | NLM:ATC:A10BJ | Glucagon-like peptide-1 (GLP-1) analogues |
|  | date constraint | | The terms in this group occurred between Jan 1, 2018 and Dec 31, 2022 | | |
|  | event relationship | | Any instance of Group 16B occurred at least 1 day before the first instance of Group 16A | | |
|  | **Group 16B No Lupus erthematosus** | | | | |
|  | cannot have |  | diagnosis | UMLS:ICD10CM:L93 | Lupus erythematosus |
| Group 17 | | | | | |
|  | **Group 17A : GLP-1 RA use** | | | | |
|  | must have |  | medication | NLM:ATC:A10BJ | Glucagon-like peptide-1 (GLP-1) analogues |
|  | date constraint | | The terms in this group occurred between Jan 1, 2018 and Dec 31, 2022 | | |
|  | event relationship | | Any instance of Group 17B occurred at least 1 day before the first instance of Group 17A | | |
|  | **Group 17B No Hidradenitis suppurativa** | | | | |
|  | cannot have |  | diagnosis | UMLS:ICD10CM:L73.2 | Hidradenitis suppurativa |
| Group 18 | | | | | |
|  | **Group 18A : GLP-1 RA use** | | | | |
|  | must have |  | medication | NLM:ATC:A10BJ | Glucagon-like peptide-1 (GLP-1) analogues |
|  | date constraint | | The terms in this group occurred between Jan 1, 2018 and Dec 31, 2022 | | |
|  | event relationship | | Any instance of Group 18B occurred at least 1 day before the first instance of Group 18A | | |
|  | **Group 18B No Systemic sclerosis** | | | | |
|  | cannot have |  | diagnosis | UMLS:ICD10CM:M34 | Systemic sclerosis [scleroderma] |
| Group 19 | | | | | |
|  | **Group 19A : GLP-1 RA use** | | | | |
|  | must have |  | medication | NLM:ATC:A10BJ | Glucagon-like peptide-1 (GLP-1) analogues |
|  | date constraint | | The terms in this group occurred between Jan 1, 2018 and Dec 31, 2022 | | |
|  | event relationship | | Any instance of Group 19B occurred at least 1 day before the first instance of Group 19A | | |
|  | **Group 19B No Pyodema gangrenosum** | | | | |
|  | cannot have |  | diagnosis | UMLS:ICD10CM:L88 | Pyoderma gangrenosum |
| Group 20 | | | | | |
|  | **Group 20A GLP** | | | | |
|  | must have |  | medication | NLM:ATC:A10BJ | Glucagon-like peptide-1 (GLP-1) analogues |
|  | date constraint | | The terms in this group occurred between Jan 1, 2018 and Dec 31, 2022 | | |
|  | event relationship | | Any instance of Group 20B occurred at least 1 day before the first instance of Group 20A | | |
|  | **Group 20B No Morphea** | | | | |
|  | cannot have |  | diagnosis | UMLS:ICD10CM:L94.0 | Localized scleroderma [morphea] |

**Query Criteria for Cohort 2 (query name: DPP-4i)**

| Group 1 | | | | | |
| --- | --- | --- | --- | --- | --- |
|  | **Visit >=3** | | | | |
|  | must have |  | visit | TNX:Visit | Visit |
|  | number of instances | | Greater than or equal to 3 instances | | |
|  | date constraint | | The terms in this group occurred at any time | | |
| Group 2 | | | | | |
|  | **Type 2 DM** | | | | |
|  | must have |  | diagnosis | UMLS:ICD10CM:E11 | Type 2 diabetes mellitus (at least 18 years old at event) |
|  | date constraint | | The terms in this group occurred between Jan 1, 2018 and Dec 31, 2022 | | |
| Group 3 | | | | | |
|  | **DPP-4i use** | | | | |
|  | must have |  | medication | NLM:ATC:A10BH | Dipeptidyl peptidase 4 (DPP-4) inhibitors |
|  | date constraint | | The terms in this group occurred between Jan 1, 2018 and Dec 31, 2022 | | |
| Group 4 | | | | | |
|  | **Group 4A : DPP-4i use** | | | | |
|  | must have |  | medication | NLM:ATC:A10BH | Dipeptidyl peptidase 4 (DPP-4) inhibitors |
|  | date constraint | | The terms in this group occurred between Jan 1, 2018 and Dec 31, 2022 | | |
|  | event relationship | | Any instance of Group 4B occurred at least 1 day before the first instance of Group 4A | | |
|  | **Group 4B No GLP-1 RA** | | | | |
|  | cannot have |  | medication | NLM:ATC:A10BJ | Glucagon-like peptide-1 (GLP-1) analogues |
| Group 5 | | | | | |
|  | **Group 5A : DPP-4i use** | | | | |
|  | must have |  | medication | NLM:ATC:A10BH | Dipeptidyl peptidase 4 (DPP-4) inhibitors |
|  | date constraint | | The terms in this group occurred between Jan 1, 2018 and Dec 31, 2022 | | |
|  | event relationship | | Any instance of Group 5B occurred at least 1 day before the first instance of Group 5A | | |
|  | **Group 5B no GLP-1 RA and DPP-4i** | | | | |
|  | cannot have |  | medication | NLM:ATC:A10BJ | Glucagon-like peptide-1 (GLP-1) analogues |
|  |  | or | medication | NLM:ATC:A10BH | Dipeptidyl peptidase 4 (DPP-4) inhibitors |
| Group 6 | | | | | |
|  | **Group 6A : DPP-4i use** | | | | |
|  | must have |  | medication | NLM:ATC:A10BH | Dipeptidyl peptidase 4 (DPP-4) inhibitors |
|  | date constraint | | The terms in this group occurred between Jan 1, 2018 and Dec 31, 2022 | | |
|  | event relationship | | Any instance of Group 6B occurred at least 1 day before the first instance of Group 6A | | |
|  | **Group 6B No Neoplasms** | | | | |
|  | cannot have |  | diagnosis | UMLS:ICD10CM:C00-D49 | Neoplasms |
| Group 7 | | | | | |
|  | **Group 7A : DPP-4i use** | | | | |
|  | must have |  | medication | NLM:ATC:A10BH | Dipeptidyl peptidase 4 (DPP-4) inhibitors |
|  | date constraint | | The terms in this group occurred between Jan 1, 2018 and Dec 31, 2022 | | |
|  | event relationship | | Any instance of Group 7B occurred at least 1 day before the first instance of Group 7A | | |
|  | **Group 7B No Transplant** | | | | |
|  | cannot have |  | diagnosis | UMLS:ICD10CM:Z94 | Transplanted organ and tissue status |
| Group 8 | | | | | |
|  | **Group 8A : DPP-4i use** | | | | |
|  | must have |  | medication | NLM:ATC:A10BH | Dipeptidyl peptidase 4 (DPP-4) inhibitors |
|  | date constraint | | The terms in this group occurred between Jan 1, 2018 and Dec 31, 2022 | | |
|  | event relationship | | Any instance of Group 8B occurred at least 1 day before the first instance of Group 8A | | |
|  | **Group 8B No Psoriasis** | | | | |
|  | cannot have |  | diagnosis | UMLS:ICD10CM:L40 | Psoriasis |
| Group 9 | | | | | |
|  | **Group 9A : DPP-4i use** | | | | |
|  | must have |  | medication | NLM:ATC:A10BH | Dipeptidyl peptidase 4 (DPP-4) inhibitors |
|  | date constraint | | The terms in this group occurred between Jan 1, 2018 and Dec 31, 2022 | | |
|  | event relationship | | Any instance of Group 9B occurred at least 1 day before the first instance of Group 9A | | |
|  | **Group 9B No Alopecia areata** | | | | |
|  | cannot have |  | diagnosis | UMLS:ICD10CM:L63 | Alopecia areata |
| Group 10 | | | | | |
|  | **Group 10A : DPP-4i use** | | | | |
|  | must have |  | medication | NLM:ATC:A10BH | Dipeptidyl peptidase 4 (DPP-4) inhibitors |
|  | date constraint | | The terms in this group occurred between Jan 1, 2018 and Dec 31, 2022 | | |
|  | event relationship | | Any instance of Group 10B occurred at least 1 day before the first instance of Group 10A | | |
|  | **Group 10B No Vitilligo** | | | | |
|  | cannot have |  | diagnosis | UMLS:ICD10CM:L80 | Vitiligo |
| Group 11 | | | | | |
|  | **Group 11A : DPP-4i use** | | | | |
|  | must have |  | medication | NLM:ATC:A10BH | Dipeptidyl peptidase 4 (DPP-4) inhibitors |
|  | date constraint | | The terms in this group occurred between Jan 1, 2018 and Dec 31, 2022 | | |
|  | event relationship | | Any instance of Group 11B occurred at least 1 day before the first instance of Group 11A | | |
|  | **Group 11B No Lichen planus** | | | | |
|  | cannot have |  | diagnosis | UMLS:ICD10CM:L43 | Lichen planus |
| Group 12 | | | | | |
|  | **Group 12A : DPP-4i use** | | | | |
|  | must have |  | medication | NLM:ATC:A10BH | Dipeptidyl peptidase 4 (DPP-4) inhibitors |
|  | date constraint | | The terms in this group occurred between Jan 1, 2018 and Dec 31, 2022 | | |
|  | event relationship | | Any instance of Group 12B occurred at least 1 day before the first instance of Group 12A | | |
|  | **Group 12B No Pemphigus** | | | | |
|  | cannot have |  | diagnosis | UMLS:ICD10CM:L10 | Pemphigus |
| Group 13 | | | | | |
|  | **Group 13A : DPP-4i use** | | | | |
|  | must have |  | medication | NLM:ATC:A10BH | Dipeptidyl peptidase 4 (DPP-4) inhibitors |
|  | date constraint | | The terms in this group occurred between Jan 1, 2018 and Dec 31, 2022 | | |
|  | event relationship | | Any instance of Group 13B occurred at least 1 day before the first instance of Group 13A | | |
|  | **Group 13B No Bullous pemphigoid** | | | | |
|  | cannot have |  | diagnosis | UMLS:ICD10CM:L12.0 | Bullous pemphigoid |
| Group 14 | | | | | |
|  | **Group 14A : DPP-4i use** | | | | |
|  | must have |  | medication | NLM:ATC:A10BH | Dipeptidyl peptidase 4 (DPP-4) inhibitors |
|  | date constraint | | The terms in this group occurred between Jan 1, 2018 and Dec 31, 2022 | | |
|  | event relationship | | Any instance of Group 14B occurred at least 1 day before the first instance of Group 14A | | |
|  | **Group 14B No Atopic dermatitis** | | | | |
|  | cannot have |  | diagnosis | UMLS:ICD10CM:L20 | Atopic dermatitis |
| Group 15 | | | | | |
|  | **Group 15A : DPP-4i use** | | | | |
|  | must have |  | medication | NLM:ATC:A10BH | Dipeptidyl peptidase 4 (DPP-4) inhibitors |
|  | date constraint | | The terms in this group occurred between Jan 1, 2018 and Dec 31, 2022 | | |
|  | event relationship | | Any instance of Group 15B occurred at least 1 day before the first instance of Group 15A | | |
|  | **Group 15B No Dermatopolymyositis** | | | | |
|  | cannot have |  | diagnosis | UMLS:ICD10CM:M33 | Dermatopolymyositis |
| Group 16 | | | | | |
|  | **Group 16A : DPP-4i use** | | | | |
|  | must have |  | medication | NLM:ATC:A10BH | Dipeptidyl peptidase 4 (DPP-4) inhibitors |
|  | date constraint | | The terms in this group occurred between Jan 1, 2018 and Dec 31, 2022 | | |
|  | event relationship | | Any instance of Group 16B occurred at least 1 day before the first instance of Group 16A | | |
|  | **Group 16B No Lupus erythematosus** | | | | |
|  | cannot have |  | diagnosis | UMLS:ICD10CM:L93 | Lupus erythematosus |
| Group 17 | | | | | |
|  | **Group 17A : DPP-4i use** | | | | |
|  | must have |  | medication | NLM:ATC:A10BH | Dipeptidyl peptidase 4 (DPP-4) inhibitors |
|  | date constraint | | The terms in this group occurred between Jan 1, 2018 and Dec 31, 2022 | | |
|  | event relationship | | Any instance of Group 17B occurred at least 1 day before the first instance of Group 17A | | |
|  | **Group 17B No Hidradenitis suppurativa** | | | | |
|  | cannot have |  | diagnosis | UMLS:ICD10CM:L73.2 | Hidradenitis suppurativa |
| Group 18 | | | | | |
|  | **Group 18A : DPP-4i use** | | | | |
|  | must have |  | medication | NLM:ATC:A10BH | Dipeptidyl peptidase 4 (DPP-4) inhibitors |
|  | date constraint | | The terms in this group occurred between Jan 1, 2018 and Dec 31, 2022 | | |
|  | event relationship | | Any instance of Group 18B occurred at least 1 day before the first instance of Group 18A | | |
|  | **Group 18B No Systemic sclerosis** | | | | |
|  | cannot have |  | diagnosis | UMLS:ICD10CM:M34 | Systemic sclerosis [scleroderma] |
| Group 19 | | | | | |
|  | **Group 19A : DPP-4i use** | | | | |
|  | must have |  | medication | NLM:ATC:A10BH | Dipeptidyl peptidase 4 (DPP-4) inhibitors |
|  | date constraint | | The terms in this group occurred between Jan 1, 2018 and Dec 31, 2022 | | |
|  | event relationship | | Any instance of Group 19B occurred at least 1 day before the first instance of Group 19A | | |
|  | **Group 19B Pyoderma gangrenosum** | | | | |
|  | cannot have |  | diagnosis | UMLS:ICD10CM:L88 | Pyoderma gangrenosum |
| Group 20 | | | | | |
|  | **Group 20A : DPP-4i use** | | | | |
|  | must have |  | medication | NLM:ATC:A10BH | Dipeptidyl peptidase 4 (DPP-4) inhibitors |
|  | date constraint | | The terms in this group occurred between Jan 1, 2018 and Dec 31, 2022 | | |
|  | event relationship | | Any instance of Group 20B occurred at least 1 day before the first instance of Group 20A | | |
|  | **Group 20B No Morphea** | | | | |
|  | cannot have |  | diagnosis | UMLS:ICD10CM:L94.0 | Localized scleroderma [morphea] |

**Outcome Definitions**

| Outcomes | Item | Code | Name |
| --- | --- | --- | --- |
| Atopic dermatitis | Diagnosis | UMLS:ICD10CM:L20 | Atopic dermatitis |
| Psoriasis | Diagnosis | UMLS:ICD10CM:L40 | Psoriasis |
| Vitiligo | Diagnosis | UMLS:ICD10CM:L80 | Vitiligo |
| Pemphigus | Diagnosis | UMLS:ICD10CM:L10 | Pemphigus |
| Bullous pemphigoid | Diagnosis | UMLS:ICD10CM:L12.0 | Bullous pemphigoid |
| Dermatomyositis | Diagnosis | UMLS:ICD10CM:M33 | Dermatopolymyositis |
| Alopecia areata | Diagnosisf | UMLS:ICD10CM:L63 | Alopecia areata |
| Lichen planus | Diagnosis | UMLS:ICD10CM:L43 | Lichen planus |
| Cutaneous lupus erythematosus | Diagnosis | UMLS:ICD10CM:L93 | Lupus erythematosus |
| Hidradenitis suppurativa | Diagnosis | UMLS:ICD10CM:L73.2 | Hidradenitis suppurativa |
| Systemic sclerosis | Diagnosis | UMLS:ICD10CM:M34 | Systemic sclerosis [scleroderma] |
| Pyoderma gangrenosum | Diagnosis | UMLS:ICD10CM:L88 | Pyoderma gangrenosum |
| Morphea | Diagnosis | UMLS:ICD10CM:L94.0 | Localized scleroderma [morphea] |
| MACE | Demographics | Deceased | Deceased |
|  | Diagnosis | UMLS:ICD10CM:I21 | Acute myocardial infarction |
|  | Diagnosis | UMLS:ICD10CM:I63 | Cerebral infarction |
| MAKE | Diagnosis | UMLS:ICD10CM:N17 | Acute kidney failure |
|  | Diagnosis | UMLS:ICD10CM:N18.6 | End stage renal disease |
|  | Procedure | UMLS:CPT:1012740 | Dialysis Services and Procedures |
|  | Procedure | UMLS:CPT:90945 | Dialysis procedure other than hemodialysis (eg, peritoneal dialysis, hemofiltration, or other continuous renal replacement therapies), with single evaluation by a physician or other qualified health care professional |
|  | Demographics | Deceased | Deceased |
| Fracture | Diagnosis | UMLS:ICD10CM:S72 | Fracture of femur |
|  | Diagnosis | UMLS:ICD10CM:M80 | Osteoporosis with current pathological fracture |
|  | Diagnosis | UMLS:ICD10CM:M81 | Osteoporosis without current pathological fracture |
| Aortic aneurysm/dissection | Diagnosis | UMLS:ICD10CM:I71.0 | Dissection of aorta |
|  | Diagnosis | UMLS:ICD10CM:I71.2 | Thoracic aortic aneurysm, without rupture |
|  | Diagnosis | UMLS:ICD10CM:I71.4 | Abdominal aortic aneurysm, without rupture |

**Codes for baseline parameters**

Propensity score matching was performed on all listed characteristics. Characteristics of the cohorts before and after matching are summarized in the table below.

|  | **Characteristics after propensity score matching** | | | | |
| --- | --- | --- | --- | --- | --- |
|  | **Demographics** | | | | |
|  | AI | Age at Index | | | |
|  | Male | Male | | | |
|  | 2106-3 | White | | | |
|  | 2054-5 | African American | | | |
|  | 2028-9 | Asian | | | |
|  | 2131-1 | Other Race | | | |
|  | **Lifestyles** | | | | |
|  | ICD-10-CM code | Name | | | |
|  | Z55-Z65 | Persons with potential health hazards related to socioeconomic and psychosocial circumstances | | | |
|  | Z59 | Problems related to housing and economic circumstances | | | |
|  | Z56 | Problems related to employment and unemployment | | | |
|  | Z72.0 | Tobacco use | | | |
|  | F17 | Nicotine dependence | | | |
|  | F10 | Alcohol related disorders | | | |
|  | Z72 | Problems related to lifestyle | | | |
|  | **Diagnosis** | | | | |
|  | ICD-10-CM code | Name | | | |
|  | E78 | Disorders of lipoprotein metabolism and other lipidemias | | | |
|  | I10-I1A | Hypertensive diseases | | | |
|  | I50 | Heart failure | | | |
|  | I48 | Atrial fibrillation and flutter | | | |
|  | I42 | Cardiomyopathy | | | |
|  | I20-I25 | Ischemic heart diseases | | | |
|  | I73.9 | Peripheral vascular disease, unspecified | | | |
|  | N17-N19 | Acute kidney failure and chronic kidney disease | | | |
|  | I35 | Nonrheumatic aortic valve disorders | | | |
|  | I60-I69 | Cerebrovascular diseases | | | |
|  | J44 | Other chronic obstructive pulmonary disease | | | |
|  | Z87.891 | Personal history of nicotine dependence | | | |
|  | M10 | Gout | | | |
|  | E00-E07 | Disorders of thyroid gland | | | |
|  | K74 | Fibrosis and cirrhosis of liver | | | |
|  | **Medication** | | | | |
|  | ATC Code | Name | | | |
|  | A10A | Insulins and analogues | | | |
|  | C10 | Lipid modifying agents | | | |
|  | C09 | Agents acting on the renin-angiotensin system | | | |
|  | C03 | Diuretics | | | |
|  | C07 | Beta blocking agents | | | |
|  | C08 | Calcium channel blockers | | | |
|  | L01 | Antineoplastic and immunomodulating agents | | | |
|  | N03 | Antiepileptics | | | |
|  | A10BB | Sulfonylureas | | | |
|  | A10BG | Thiazolidinediones | | | |
|  | A10BF | Alpha glucosidase inhibitors | | | |
|  | H02A | Corticosteroids for systemic use, plain | | | |
|  | A10BA | Biguanides | | | |
|  | A10BK | Sodium-glucose co-transporter 2 (SGLT2) inhibitors | | | |
|  | R06 | Antihistamines for systemic use | | | |
|  | **Laboratory** | | | | |
|  | TNX-curated | Name | Unit | Missing rate after PSM matching | |
|  |  | | | GLP-1 RA users | DPP-4i users |
|  | 9044 | Alanine aminotransferase | U/L | 43.00% | 43.30% |
|  | 9047 | Aspartate aminotransferase | U/L | 43.20% | 43.50% |
|  | 9004 | Triglyceride | mg/dL | 53.10% | 53.40% |
|  | 9083 | BMI | kg/m^2^ | 39.60% | 39.40% |
|  | 9037 | Hemoglobin A1c | % | 43.80% | 44.80% |
|  | 9002 | Cholesterol in LDL | mg/dL | 54.00% | 54.30% |
|  | 9001 | Cholesterol in HDL | mg/dL | 52.90% | 53.10% |
|  | 9014 | Hemoglobin | g/dL | 45.10% | 45.40% |
|  | 9045 | Albumin | g/dL | 44.00% | 44.30% |
|  | 8001 | Glomerular filtration rate (MDRD) | mmol/L | 37.40% | 37.20% |
|  | 9029 | Sodium | mmol/L | 36.10% | 36.40% |
|  | 9028 | Potassium | mmol/L | 37.00% | 37.40% |
|  | 9022 | Calcium | mg/dL | 36.90% | 37.20% |
|  | 9027 | Phosphate | mg/dL | 87.70% | 87.90% |
|  | 14959-1 | Microalbumin/Creatinine | mg/g{creat} | 85.20% | 85.40% |
|  | 9085 | Blood Pressure, Systolic | mm[Hg] | 36.70% | 37.00% |
|  | 9086 | Blood Pressure, Diastolic | mm [Hg] | 36.70% | 37.00% |

**Method S2.** The details of propensity score matching in the TriNetX platform

**(https://support.trinetx.com/hc/en-us/articles/360011978033)**

To conduct PSM using the TNX Research platform, you must first identify two cohorts of interest, index events, outcomes of interest, and attributes of patients which may act as confounders to the outcomes of interest. For the purpose of this article, these attributes will be called “covariates.”

Within Balance, when you run a propensity score matching analysis, the system conducts a propensity score matching to balance the cohorts:

1. For each patient in each cohort, the system computes values for each covariate.
2. These data form a matrix of covariate values for each
3. The system performs a logistic regression on the pooled matrices, to “predict” which cohort each patient originates from. The value of this model for a patient is that patient’s predicted probability of being in the second cohort or “propensity score.”
4. For each patient in the smaller cohort, the system chooses as match from the larger cohort (if any patients in the larger cohort are close enough). The pairs then form a subset of each cohort.

Within Outcomes, when you run compare cohorts after matching, the system conducts a propensity score matching to compare outcomes between the balance the cohorts:

1. For each patient in each cohort, the system computes both the outcome(s) of interest and values for each covariate.
2. These data form a matrix of covariate values for each
3. The system performs a logistic regression on the pooled matrices, to “predict” which cohort each patient originates from. The value of this model for a patient is that patient’s predicted probability of being in the second cohort or “propensity score.”
4. For each patient in the smaller cohort, the system chooses as match from the larger cohort (if any patients in the larger cohort are close enough). The pairs then form a subset of each cohort.
5. The system compares outcomes on these after matching subsets, rather than the original cohorts.

**Values in the Covariate Matrix**

In the covariate matrix, each row represents one patient, and each column represents one covariate. Each cell contains exactly one non-null numerical value.

 All covariates are one of the following forms:

- **Binary:** yes/no;
- **Categorical:** real values placed in categories based on their value; or
- **Continuous:** real values represented directly.

When a covariate is binary (for example, a patient having history of asthma), the cell for that patient and covariate is set to 0 for “not present” or 1 for “is present.”

When a covariate is categorical, the categories are specified as ranges that the continuous variable can take (for example, having a value for a Sodium lab between 130 and 140). Each category becomes a distinct covariate which is either 0 for “not present” or 1 for “is present.” It is possible for all columns to be 0 (for example if there are no values in the time window, or if all values are outside all the categories). It is also possible for multiple values to be 1 (for example if there are multiple values in the time window, or if the categories overlap).

When a covariate is continuous (for example, current age, or age at time of the index event), then the value for that patient (if any) is placed in the relevant cell.

**Details on Scoring and Logistic Regression**

When you run propensity score matching, the system generates a propensity score for each patient in each cohort. The propensity score ranges between 0 and 1 and indicates the predicted probability a patient is in cohort B given the patient’s covariates.

To uses logistic regression to generate the propensity scores, through an implementation of the well-tested, standard software package [scikit-learn](https://scikit-learn.org/). The code used in production is as follows (some logging, etc. removed for clarity):

import numpy as np

from sklearn.linear_model import LogisticRegression

from sklearn.preprocessing import Imputer

from impute import fill_nans

def fill_nans(data, axis=0):

  imp = Imputer(missing_values='NaN', strategy=method, axis=axis)

    return imp.fit_transform(data)

def propensity_scores(matrix_a, matrix_b):

matrix_a = fill_nans(matrix_a, method='mean')

matrix_b = fill_nans(matrix_b, method='mean')

full_matrix = np.concatenate((matrix_a, matrix_b), axis=0)

# cohort A is "zero" and cohort B is "one" from the regression’s perspective

target_a = np.zeros(matrix_a.shape[0], dtype=float)

target_b = np.ones(matrix_b.shape[0], dtype=float)

target = np.concatenate([target_a, target_b])

lr = LogisticRegression(C=1000)

lr.fit(full_matrix, target)

def get_scores(X):

# .predict_proba returns [prob_class_zero, prob_class_one] for each row,

# while the score we want is the probability of being in class B

full_scores = lr.predict_proba(X)

return full_scores[:, 1]

return (get_scores(matrix_a), get_scores(matrix_b))

Here the “score” for each patient is the regression model applied to that patient’s row. Informally, this is the probability that the patient belongs to cohort B, based on the cohorts it has seen.

Here “matrix_a” is the covariate matrix for the cohort you selected as Cohort A. Likewise, “matrix_b” is the covariate matrix for the cohort you selected as Cohort B.  Data are pooled across all HCOs, so all patients in the analysis are a row in one of these matrices. Note that the code instructs the system to replace all missing (NaN) values in each matrix with the mean from that column; however, at time of writing all covariates are either binary, categorical (which expands to a set of binary columns), or continuous but guaranteed to exist, so this imputation is vacuous.

The call “LogisticRegression(C=1000)” means we predict the probability of being in cohort B using logistic regression. The value of C means we weight the mean residual by a factor of 1000 as compared to the norm of the coefficients. This means we are using a very small amount of L2 (ridge) regression, which is needed to make the objective function convex (so the regression converges), but with such a small value, it has very little effect on the model. Due to the very small amount of regularization and the scale of our covariates being constrained, it is not necessary to perform mean normalization as a preprocessing step.

**Matching Details**

Once the system has generated a propensity score for each patient, the system performs matching to identify the matched subsets. We use “greedy nearest neighbor matching” with a caliper of 0.1 pooled standard deviations. Sample code is here:

import numpy as np

def _pooled_sd(floats_a, floats_b):

var_a = np.var(floats_a)

var_b = np.var(floats_b)

return sqrt((var_a + var_b) / 2.0)

def _naive_nearest_neighbor_match(scores_a, scores_b, caliper):

# Perform nearest neighbor matching. Assume that len(scores_a) <=len(scores_b)

num_a, num_b = len(scores_a), len(scores_b)

max_diff = _pooled_sd(scores_a, scores_b) * caliper

chosen_a = np.zeros(num_a, dtype=bool) # True if the patient is matched, start false

chosen_b = np.zeros(num_b, dtype=bool)

for a_ind in range(0, num_a):

best_diff = max_diff

best_b_ind = None

found_any = False

for b_ind in range(0, num_b):

if chosen_b[b_ind]:

continue

diff = abs(scores_a[a_ind] - scores_b[b_ind])

if diff < best_diff or (diff == best_diff and not found_any):

best_diff = diff

best_b_ind = b_ind

found_any = True

if best_b_ind is not None:

chosen_a[a_ind] = True

chosen_b[best_b_ind] = True

return (chosen_a, chosen_b)

*Note:* the code used in production is highly optimized and significantly longer. The above code is taken directly from our test fixtures and is used to ensure that the optimized code produces identical results to the above simplified code.

We use a “caliper” of 0.1 pooled standard deviations of the propensity scores in aggregate, which means that patients with very different propensity scores are not matched. The system matches patients using the following algorithm:

1. For each patient in cohort A (assumed to be the smaller of the two), and identify the patient whose score is closest to the patient in cohort A from the patients in cohort B who have not yet been matched.
2. If a match is found, mark both as “chosen” and move on.
3. At the end, return the labels of each cohort, indicating which patients were chosen and which were not. The patients who were chosen form the “matched” cohorts.

**Pooling the Cohorts, and Compensating for Unbalanced Cohorts across HCOs**

TriNetX pulls data from a federated data network made up of many healthcare organizations across the world. Each site computes a covariate matrix for the patients they contribute to the analysis and send it to a central processing point to be pooled and analyzed as a single matrix.

The order of the rows in the matrix should not impact the propensity scores generated for each patient; logistic regression is highly stable with respect to permuting the rows.  In contrast, nearest neighbor matching, can be influenced by the order of rows in the matrix. For example, if two identical patients in cohort B match equally well to a patient in cohort A, the first will be chosen and the second will not.  Therefore, if the order of the rows carries some information (perhaps as an artifact of the pooling), the order can introduce bias.

To eliminate this bias, we randomize the order of the records in the covariate matrix.  We sort the provider results by the unique ID of the HCO (so the information of “which HCO responded first” is deleted), concatenate the matrices into one large matrix, then shuffle them using np.random.shuffle(full_matrix_a) and np.random.shuffle(full_matrix_b). To assure determinism, a call to np.random.seed(FIXED_SEED) precedes all calls to shuffle, so that successive runs do not change unless the underlying data changes.

**Figure S1.** The distribution of follow-up time

Abbreviations: GLP1-RA, glucagon-like peptide-1 receptor agonist. DPP-4i, dipeptidyl peptidase 4 inhibitor.


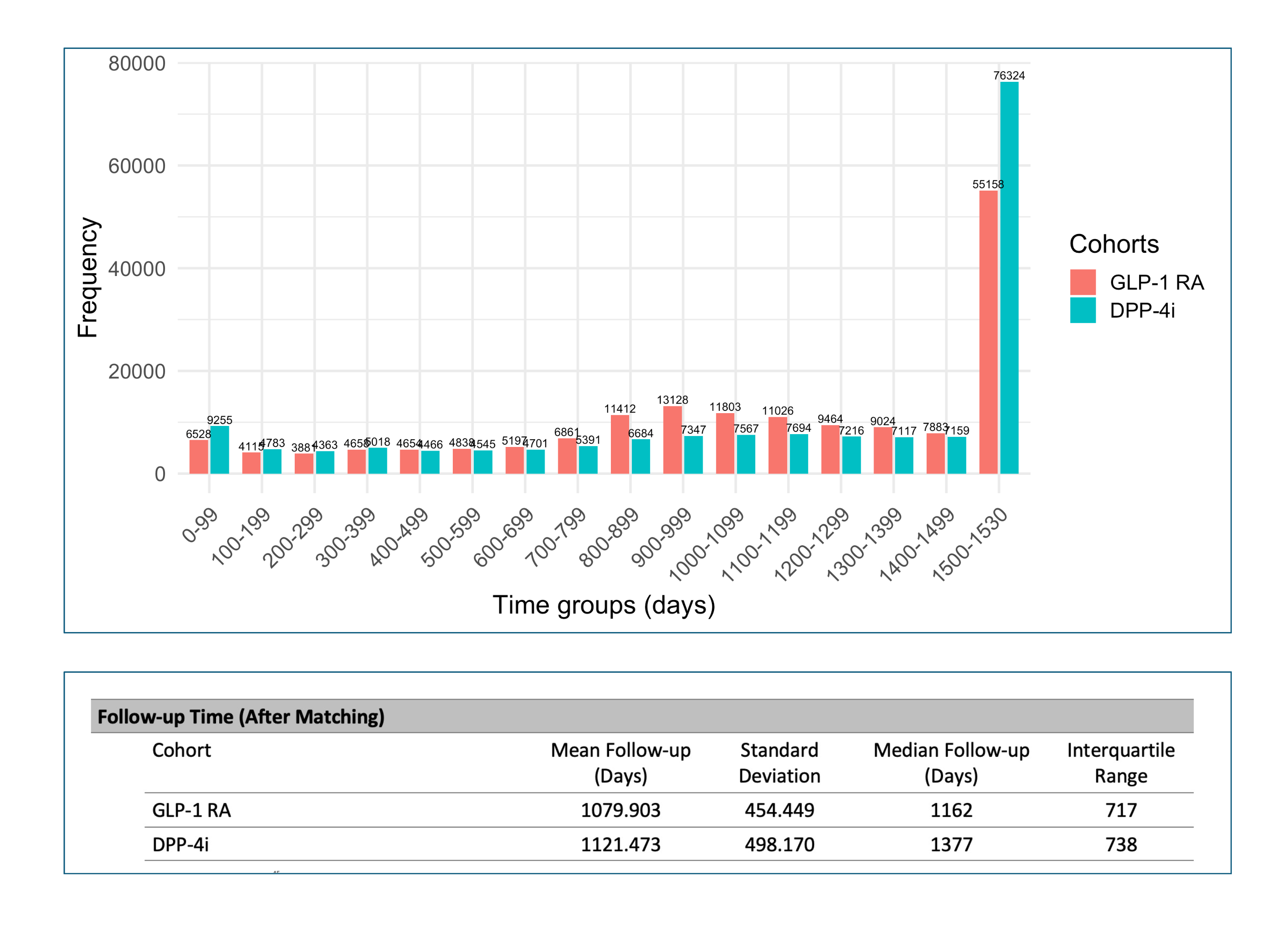


**Figure S2****.** Kaplan-Meier cumulative event-free plots

comparing (A) psoriasis, (B) pemphigus, and (C) bullous pemphigoid in the study population between patients receiving GLP1-RAs and DPP-4is.

Abbreviations: GLP1-RA, glucagon-like peptide receptor agonist. DPP-4i, dipeptidyl peptidase 4 inhibitors.

**
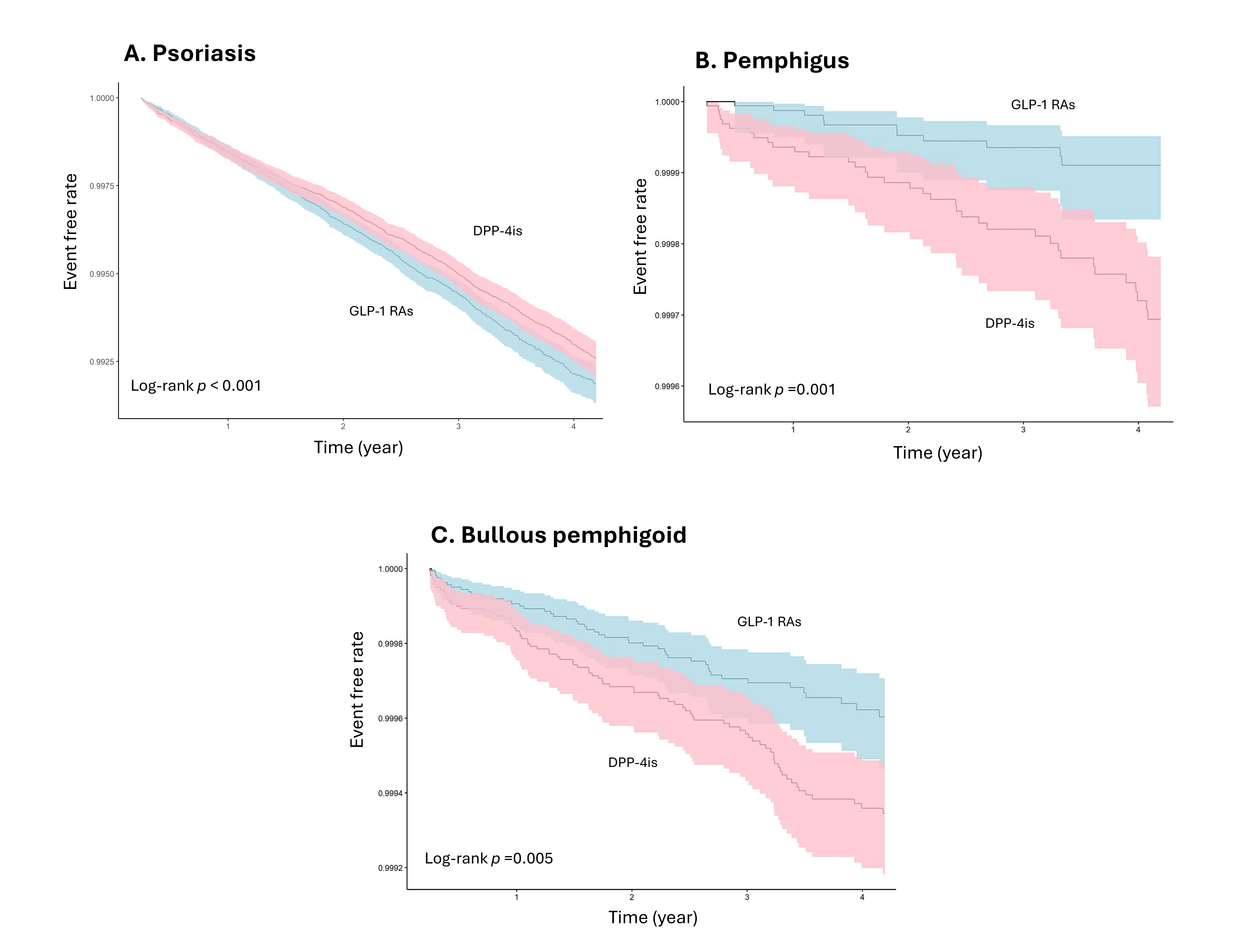
**

**Figure S3.** Subgroup on the outcome of Psoriasis

Outcomes of psoriasis between patients receiving GLP-1 RAs versus DPP-4is were evaluated.

**
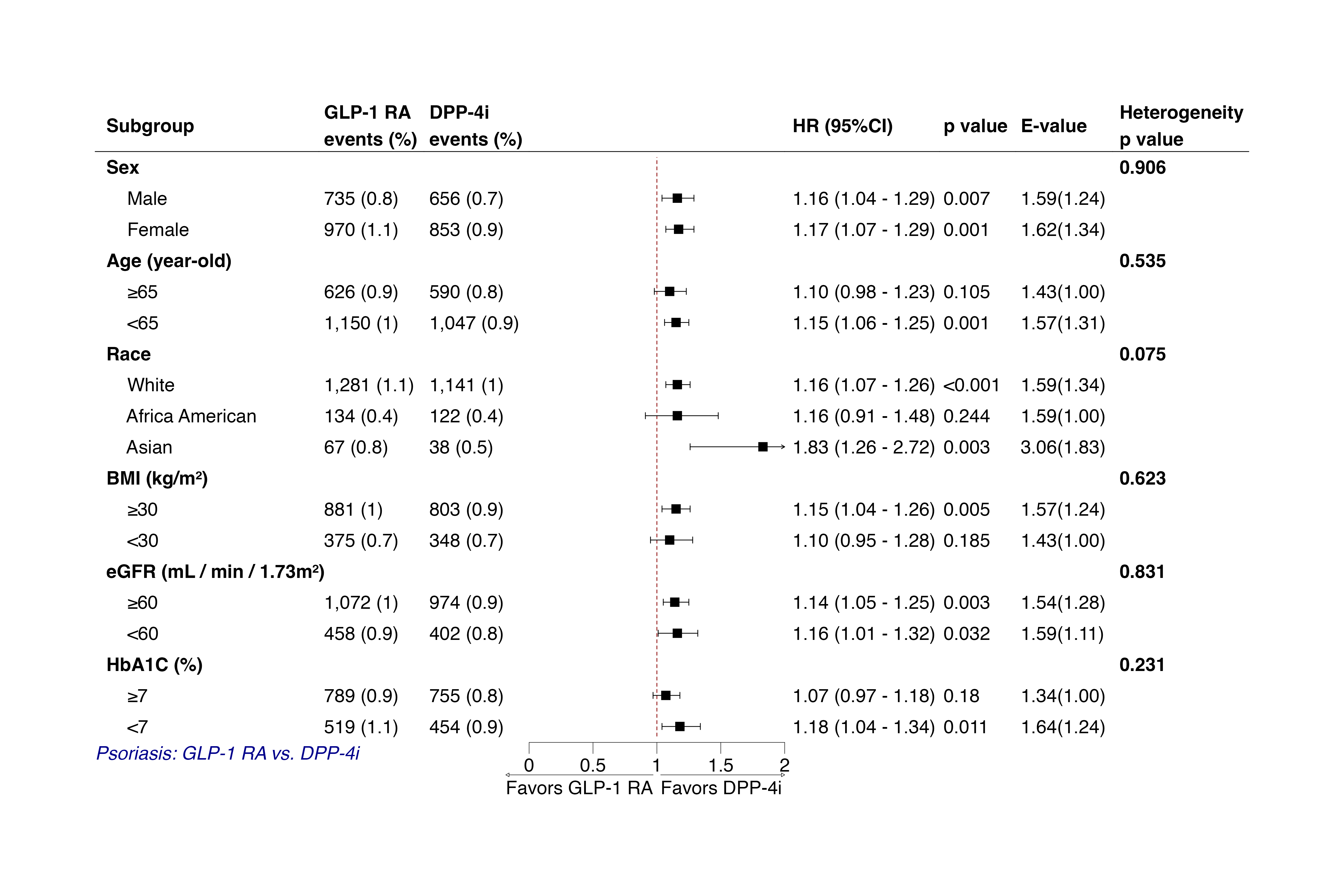
**Abbreviations: GLP1-RA, glucagon-like peptide-1 receptor agonist. DPP-4i, dipeptidyl peptidase 4 inhibitor.

**Figure S4.** Subgroup on the outcome of pemphigus

Outcomes of pemphigus between patients receiving GLP-1 RAs versus DPP-4is were evaluated.

**
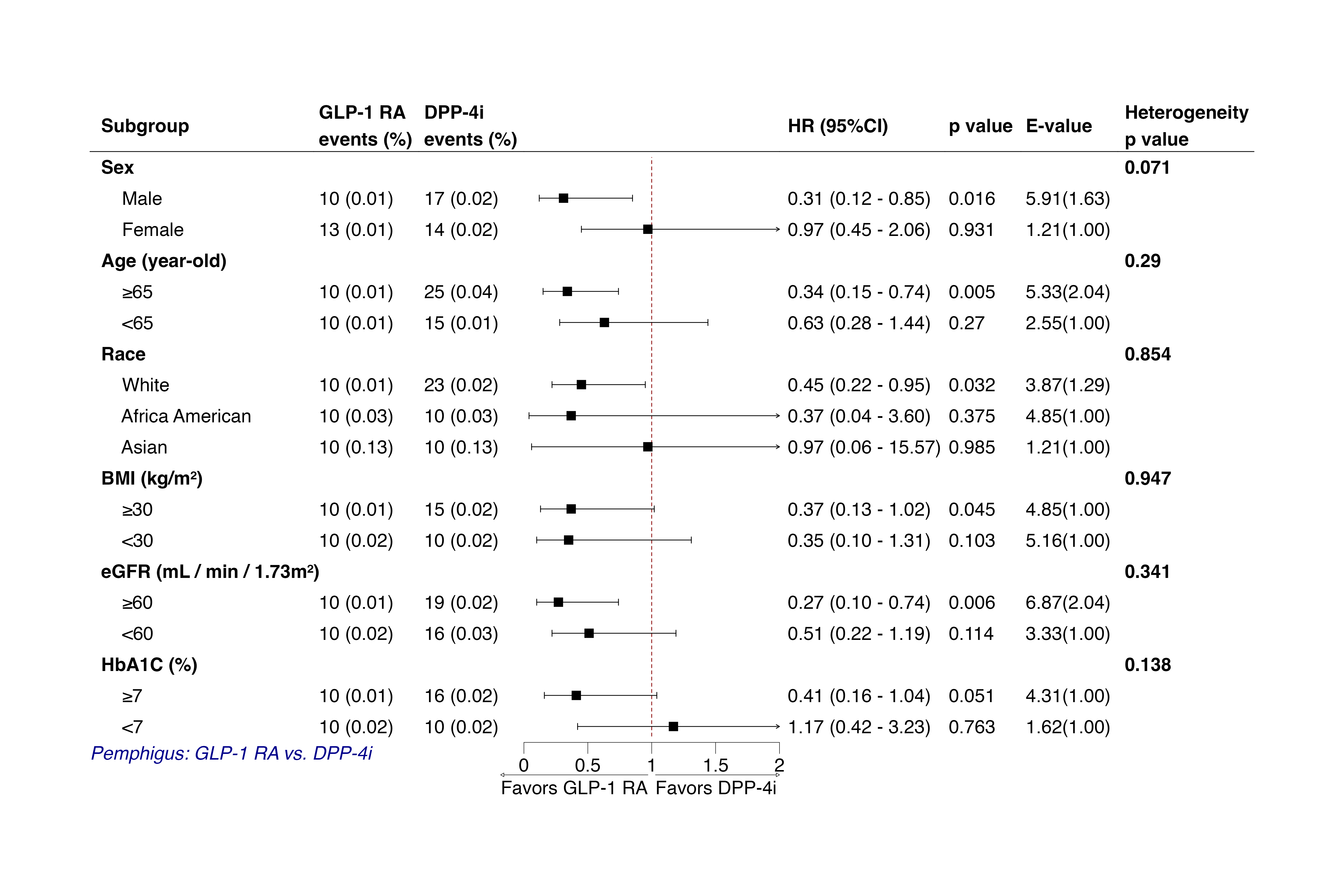
**Abbreviations: GLP1-RA, glucagon-like peptide-1 receptor agonist. DPP-4i, dipeptidyl peptidase 4 inhibitor.

**Figure S5.** Subgroup on the outcome of bullous pemphigoid

Outcomes of bullous pemphigoid between patients receiving GLP-1 RAs versus DPP-4is were evaluated.

Abbreviations: GLP1-RA, glucagon-like peptide-1 receptor agonist. DPP-4i, dipeptidyl peptidase 4 inhibitor.


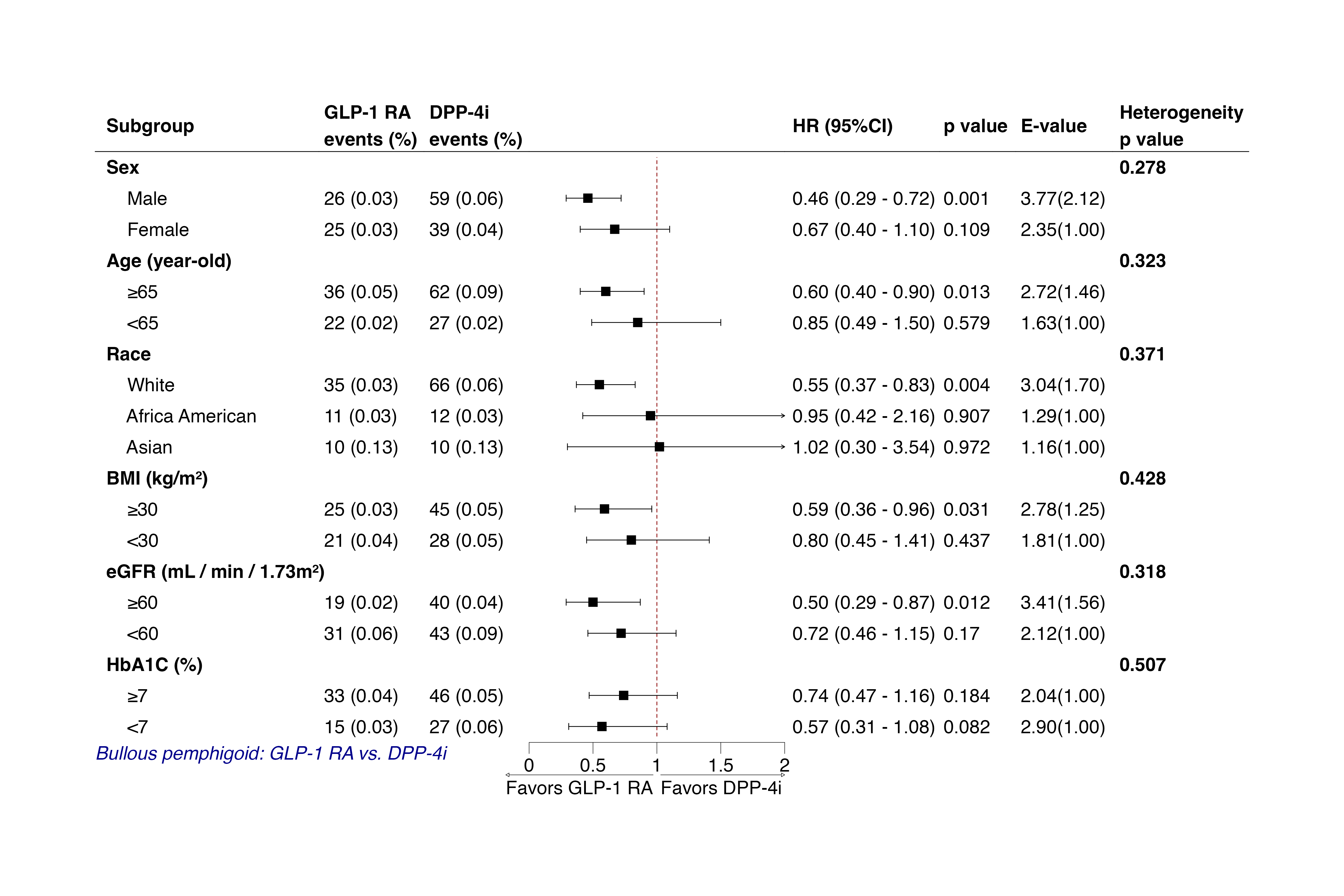


| **Table S1.** Target trail emulation | | |
| --- | --- | --- |
| **Component** | **Target trial (hypothetical pragmatic RCT)** | **Target trial emulation (observational implementation)** |
| **Research question** | Among adults with type 2 diabetes mellitus (T2DM), what is the effect of initiating a GLP‑1 receptor agonist (GLP‑1 RA) vs initiating a DPP‑4 inhibitor (DPP‑4i) on incident dermatologic outcomes? | Same question using de-identified EHR data from the TriNetX US Collaborative Network. |
| **Eligibility criteria** | Adults (≥18 years) with T2DM, eligible to initiate either treatment; adequate baseline history; no prior use of study drugs; free of study outcomes at baseline. | Adults (≥18 years) with T2DM with encounters between 2018-01-01 and 2022-12-31, and ≥3 prior healthcare visits; new-user requirement: no GLP‑1 RA or DPP‑4i exposure in the 6 months prior to index; excluded if history of malignancy or organ transplantation; excluded if pre-existing dermatologic conditions of interest at baseline (atopic dermatitis, psoriasis, vitiligo, pemphigus, bullous pemphigoid, dermatomyositis, alopecia areata, lichen planus, cutaneous lupus erythematosus, hidradenitis suppurativa, systemic sclerosis, pyoderma gangrenosum, morphea). |
| **Treatment strategies (interventions)** | Initiate GLP‑1 RA at baseline vs initiate DPP‑4i at baseline. | Initiation defined by first recorded prescription during the study period; DPP‑4i selected as an active comparator to reduce confounding by indication (similar clinical indication/timing). |
| **Treatment assignment** | Random assignment at baseline. | Non-random assignment addressed using propensity score matching (1:1) to balance baseline covariates. |
| **Time zero (baseline/index date)** | Date of randomization and treatment initiation. | Index date = first prescription date of GLP‑1 RA or DPP‑4i during the study period. |
| **Exposure definition / analysis approach** | Intention-to-treat (analyze as assigned regardless of switching). | Intention-to-treat emulation: analyze according to initial drug class at index, regardless of subsequent treatment changes. |
| **Lag / washout** | Pre-specified latency period to reduce reverse causation. | 3-month exposure lag after initiation; events occurring during lag not counted (to reduce protopathic/reverse-causation bias). Follow-up begins after the lag. |
| **Outcomes** | Incident dermatologic diseases during follow-up. | Incident psoriasis, pemphigus, bullous pemphigoid, atopic dermatitis, vitiligo, dermatomyositis, alopecia areata, lichen planus, cutaneous lupus erythematosus, hidradenitis suppurativa, systemic sclerosis, pyoderma gangrenosum, morphea; identified using ICD codes (Supplementary Method S1). |
| **Follow-up** | From end of lag until outcome, death, loss to follow-up, or administrative end. | From end of 3-month lag until first outcome, loss to follow-up, death, or end of study; maximum follow-up 4 years. |
| **Causal contrast (estimand)** | Effect of assignment to GLP‑1 RA initiation vs DPP‑4i initiation on time-to-incident dermatologic outcomes (hazard ratio). | Same estimand under the emulated intention-to-treat strategy. |
| **Statistical analysis** | Time-to-event models (e.g., Cox), ITT estimand; multiplicity control if multiple outcomes. | Kaplan–Meier and log-rank tests; Cox proportional hazards models to estimate HRs and 95% CIs; proportional hazards checked using generalized Schoenfeld methods in-platform; multiplicity controlled using Benjamini–Hochberg FDR (0.05) across dermatologic outcomes; missing data not imputed. |
| **Implementation details (confounding control)** | Balance baseline prognostic factors through randomization. | Propensity scores via logistic regression including demographics (age, sex, race), comorbidities, medication use, and laboratory measures (Table 1); 1:1 greedy nearest-neighbor matching with caliper 0.1 SD of logit(PS); balance assessed by SMD (<0.1).  Sensitivity analyses: positive (MACE/MAKE) and negative (glaucoma/fracture) controls; lag 3→6 months; stepwise PS models; varied follow-up windows; alternative comparators (insulin, biguanides, SGLT2is, thiazolidinediones). |
| **Cohort size (as implemented)** | Determined by trial enrollment. | Pre-matching: 288,812 GLP‑1 RA initiators; 218,716 DPP‑4i initiators. Post-matching: 169,630 per group. |
| **abbreviations:** GLP‑1 RA, glucagon-like peptide‑1 receptor agonist; DPP‑4i, dipeptidyl peptidase‑4 inhibitor; T2DM, type 2 diabetes mellitus; EHR, electronic health record; ITT, intention-to-treat; PS, propensity score; SMD, standardized mean difference; SD, standard deviation; HR, hazard ratio; CI, confidence interval; ICD, International Classification of Diseases; FDR, false discovery rate; MACE, major adverse cardiovascular event(s); MAKE, major adverse kidney event(s); SGLT2i, sodium–glucose cotransporter 2 inhibitor. | | |

| **Table S2.** The positive and negative outcome control | | | | | | |
| --- | --- | --- | --- | --- | --- | --- |
| Clinical Outcomes | GLP-1 RA  (n = 169,263) | | DDP-4i  (n = 169,263) | | GLP-1 RA vs. DDP-4 | |
|  | Events | Risk (%) | Events | Risk (%) | HR (95% CI) | *p* value |
| **Positive control** |  |  |  |  |  |  |
| MACE | 23,089 | 13.64 | 29,358 | 17.35 | 0.79 (0.78–0.80) | <0.001 |
| MAKE | 19,052 | 11.26 | 22,906 | 13.53 | 0.85 (0.83–0.86) | <0.001 |
| **Negative control** |  |  |  |  |  |  |
| Glaucoma | 7,867 | 4.65 | 7,742 | 4.57 | 1.03 (1.00–1.06) | 0.101 |
| Fracture | 7,637 | 4.51 | 7,889 | 4.66 | 0.99 (0.96–1.02) | 0.416 |
| MACE includes acute myocardial infarction, stroke and death. MAKE includes acute kidney injury, end stage of kidney disease, and death.  Abbreviation: GP1-RA, glucagon-like peptide-1 receptor agonist; DPP-4i, Dipeptidyl peptidase 4 inhibitors; HR, hazard ratio; 95% CI, 95% confidence interval, MACE, Major Adverse Cardiovascular Events; MAKE, Major Adverse Kidney Events. | | | | | | |
|  | | | | | | |

| **Table S3.** Sensitivity test by extending the index date by 6 months | | | | | | |
| --- | --- | --- | --- | --- | --- | --- |
| Clinical Outcomes | GLP-1 RA  (n = 158,228) | | DDP-4i  (n = 158,228) | | GLP-1 RA vs. DDP-4i | |
|  | Events | Risk (%) | Events | Risk (%) | HR (95% CI) | *p* value |
| Psoriasis | 1,677 | 0.899 | 1,470 | 0.788 | 1.19 (1.11–1.27) | **<0.001** |
| Pemphigus | 16 | 0.009 | 33 | 0.018 | 0.51 (0.28–0.93) | **0.024** |
| Bullous pemphigoid | 58 | 0.031 | 91 | 0.049 | 0.66 (0.48–0.92) | **0.014** |
| Atopic dermatitis | 997 | 0.535 | 911 | 0.488 | 1.15 (1.05–1.26) | **0.002** |
| Vitiligo | 133 | 0.071 | 132 | 0.071 | 1.05 (0.82–1.33) | 0.706 |
| Dermatomyositis | 77 | 0.041 | 72 | 0.039 | 1.11 (0.80–1.53) | 0.538 |
| Alopecia areata | 101 | 0.054 | 89 | 0.048 | 1.20 (0.90–1.60) | 0.207 |
| Lichen planus | 177 | 0.095 | 176 | 0.094 | 1.05 (0.85–1.30) | 0.634 |
| Cutaneous lupus erythematosus | 103 | 0.055 | 112 | 0.06 | 0.93 (0.71–1.22) | 0.591 |
| Hidradenitis suppurativa | 371 | 0.199 | 390 | 0.209 | 1.00 (0.87–1.16) | 0.978 |
| Systemic sclerosis | 83 | 0.045 | 88 | 0.047 | 0.97 (0.72–1.31) | 0.828 |
| Pyoderma gangrenosum | 28 | 0.015 | 32 | 0.017 | 0.93 (0.56–1.55) | 0.782 |
| Morphea | 86 | 0.046 | 67 | 0.036 | 1.35 (0.98–1.86) | 0.065 |
| Abbreviation: GP1-RA, glucagon-like peptide-1 receptor agonist; DPP-4i, Dipeptidyl peptidase 4 inhibitors; HR, hazard ratio; 95% CI, 95% confidence interval | | | | | | |

| **Table S4.** Models for confounding adjustment | | | | | | |
| --- | --- | --- | --- | --- | --- | --- |
| Clinical Outcomes | Model 1 | | Model 2 | | Model 3 | |
|  | HR (95% CI) | *p* value | HR (95% CI) | *p* value | HR (95% CI) | *p* value |
| Psoriasis | 1.16 (1.08–1.24) | **<0.001** | 1.16 (1.08–1.24) | **<0.001** | 1.15 (1.08–1.23) | **<0.001** |
| Pemphigus | 0.51 (0.3–0.86) | **0.009** | 0.44 (0.25–0.76) | **0.002** | 0.43 (0.24–0.79) | **0.005** |
| Bullous pemphigoid | 0.53 (0.38–0.73) | **<0.001** | 0.56 (0.4–0.78) | **<0.001** | 0.66 (0.48–0.9) | **0.009** |
| Atopic dermatitis | 1.08 (0.99–1.17) | 0.074 | 1.13 (1.04–1.23) | **0.004** | 1.17 (1.07–1.28) | **<0.001** |
| Vitiligo | 1.02 (0.81–1.28) | 0.883 | 0.97 (0.77–1.22) | 0.767 | 0.96 (0.76–1.21) | 0.724 |
| Dermatomyositis | 1.1 (0.83–1.48) | 0.505 | 1.18 (0.89–1.57) | 0.261 | 1.03 (0.76–1.39) | 0.850 |
| Alopecia areata | 1.13 (0.87–1.48) | 0.361 | 1.12 (0.85–1.47) | 0.416 | 1.12 (0.85–1.49) | 0.417 |
| Lichen planus | 1.07 (0.88–1.3) | 0.494 | 1.08 (0.9–1.31) | 0.407 | 1.09 (0.9–1.33) | 0.385 |
| Cutaneous lupus erythematosus | 0.91 (0.71–1.16) | 0.429 | 0.96 (0.75–1.23) | 0.732 | 0.89 (0.69–1.15) | 0.359 |
| Hidradenitis suppurativa | 1.1 (0.96–1.26) | 0.156 | 1.07 (0.94–1.23) | 0.323 | 1 (0.88–1.15) | 0.951 |
| Systemic sclerosis | 0.79 (0.59–1.05) | 0.106 | 0.82 (0.62–1.09) | 0.179 | 0.93 (0.7–1.24) | 0.626 |
| Pyoderma gangrenosum | 0.87 (0.55–1.37) | 0.542 | 0.84 (0.52–1.35) | 0.466 | 0.78 (0.48–1.28) | 0.329 |
| Morphea | 1.33 (0.99–1.79) | 0.062 | 1.27 (0.94–1.71) | 0.124 | 1.34 (0.99–1.82) | 0.060 |
| Model 1 adjusts for age, sex, race, lifestyles, body mass index, blood pressure, and economic circumstances in the propensity score matching. Model 2 includes Model 1 plus baseline comorbidities, while Model 3 adds baseline medication use to Model 2 in the propensity score matching. Abbreviation: HR, hazard ratio; 95% CI, 95% confidence interval. | | | | | | |

| **Table S5.** Segmenting the follow-up into three periods | | | | | | |
| --- | --- | --- | --- | --- | --- | --- |
| Clinical Outcomes | GLP-1 RA versus DPP-4i | | | | | |
|  | 3 months to 1 years | | 3 months to 2 years | | 3 months to 3 years | |
|  | HR (95% CI) | *p* value | HR (95% CI) | *p* value | HR (95% CI) | *p* value |
| Psoriasis | 1.18 (1.06–1.31) | **0.002** | 1.18 (1.08–1.28) | **<0.001** | 1.17 (1.09–1.26) | **<0.001** |
| Pemphigus | 0.39 (0.12–1.25) | 0.100 | 0.47 (0.22–0.99) | **0.041** | 0.44 (0.22–0.87) | **0.015** |
| Bullous pemphigoid | 0.5 (0.3–0.83) | **0.006** | 0.59 (0.39–0.88) | **0.009** | 0.68 (0.48–0.96) | **0.025** |
| Atopic dermatitis | 1.02 (0.88–1.18) | 0.811 | 1.09 (0.98–1.22) | 0.126 | 1.11 (1.01–1.22) | **0.026** |
| Vitiligo | 0.87 (0.59–1.28) | 0.478 | 0.98 (0.74–1.3) | 0.869 | 0.97 (0.75–1.25) | 0.817 |
| Dermatomyositis | 1.29 (0.83–2) | 0.260 | 1.09 (0.75–1.57) | 0.655 | 1.02 (0.74–1.4) | 0.927 |
| Alopecia areata | 0.93 (0.58–1.47) | 0.743 | 1.01 (0.71–1.45) | 0.952 | 1.16 (0.86–1.56) | 0.330 |
| Lichen planus | 1.06 (0.75–1.49) | 0.755 | 1.07 (0.83–1.39) | 0.588 | 1.06 (0.85–1.33) | 0.617 |
| Cutaneous lupus erythematosus | 0.96 (0.66–1.4) | 0.832 | 0.95 (0.71–1.27) | 0.706 | 0.96 (0.74–1.25) | 0.743 |
| Hidradenitis suppurativa | 0.98 (0.77–1.25) | 0.857 | 0.99 (0.83–1.19) | 0.947 | 1.02 (0.87–1.19) | 0.824 |
| Systemic sclerosis | 0.88 (0.58–1.32) | 0.529 | 0.82 (0.58–1.15) | 0.241 | 0.89 (0.66–1.21) | 0.461 |
| Pyoderma gangrenosum | 0.49 (0.22–1.09) | 0.074 | 0.67 (0.35–1.29) | 0.223 | 0.97 (0.56–1.68) | 0.910^#^ |
| Morphea | 1.6 (0.94–2.72) | 0.080 | 1.34 (0.88–2.03) | 0.172 | 1.37 (0.98–1.91) | 0.066 |
| ^#^This indicates that the proportional hazard assumption is violated.  Abbreviation: GP1-RA–glucagon-like peptide-1 receptor agonist; DPP-4i–Dipeptidyl peptidase 4 inhibitors; HR–hazard ratio; CI, confidence interval | | | | | | |
